# Areas of high conservation value at risk by plant invaders in Georgia under climate change

**DOI:** 10.1002/ece3.4005

**Published:** 2018-04-02

**Authors:** Daniel Slodowicz, Patrice Descombes, David Kikodze, Olivier Broennimann, Heinz Müller‐Schärer

**Affiliations:** ^1^ Department of Ecology and Evolution University of Fribourg Fribourg Switzerland; ^2^ Swiss Federal Research Institute WSL Birmensdorf Switzerland; ^3^ Landscape Ecology Institute of Terrestrial Ecosystems ETH Zürich Zürich Switzerland; ^4^ Institute of Botany Ilia State University Tbilisi Georgia; ^5^ Department of Ecology and Evolution University of Lausanne Lausanne Switzerland; ^6^ Institute of Earth Surface Dynamics University of Lausanne Lausanne Switzerland

**Keywords:** Caucasus, endemic plants, invasive alien plants, protected areas, species distribution models, species richness

## Abstract

Invasive alien plants (IAP) are a threat to biodiversity worldwide. Understanding and anticipating invasions allow for more efficient management. In this regard, predicting potential invasion risks by IAPs is essential to support conservation planning into areas of high conservation value (AHCV) such as sites exhibiting exceptional botanical richness, assemblage of rare, and threatened and/or endemic plant species. Here, we identified AHCV in Georgia, a country showing high plant richness, and assessed the susceptibility of these areas to colonization by IAPs under present and future climatic conditions. We used actual protected areas and areas of high plant endemism (identified using occurrences of 114 Georgian endemic plant species) as proxies for AHCV. Then, we assessed present and future potential distribution of 27 IAPs using species distribution models under four climate change scenarios and stacked single‐species potential distribution into a consensus map representing IAPs richness. We evaluated present and future invasion risks in AHCV using IAPs richness as a metric of susceptibility. We show that the actual protected areas cover only 9.4% of the areas of high plant endemism in Georgia. IAPs are presently located at lower elevations around the large urban centers and in western Georgia. We predict a shift of IAPs toward eastern Georgia and higher altitudes and an increased susceptibility of AHCV to IAPs under future climate change. Our study provides a good baseline for decision makers and stakeholders on where and how resources should be invested in the most efficient way to protect Georgia's high plant richness from IAPs.

## 1 INTRODUCTION

Plant invasions have become an increasing threat to agriculture, human health, and local biodiversity (Pimentel, Zuniga, & Morrison, 2005; Richter et al., 2013). Because invasive alien plants (IAPs) have also become a major issue in protected areas (PAs; Foxcroft, Pyšek, Richardson, & Genovesi, 2013), there is an urgent need to implement conservation actions to limit the impact of IAPs in areas of high conservation values (AHCV) such as sites exhibiting exceptional botanical richness, assemblage of rare, and threatened and/or endemic plant species. While some countries have already established procedures for the management of IAPs (e.g., USA, Switzerland; Bohren, 2006; Van Driesche, Blossey, Hoddle, Lyon, & Reardon, 2002), many other countries are still in the process of evaluating the threats and risks caused by IAPs.

Prevention of biological invasion at the earliest stage is more effective than attempts at management of well‐established infestations (Leung et al., 2002). In this regard, statistical models such as species distribution models (SDMs; Guisan & Thuiller, 2005) may represent useful tools when implementing conservation actions at large and local scales (e.g., Descombes et al., 2016; Heinänen, Erola, & von Numers, 2012). SDMs relate environmental characteristics (e.g., climatic and topographic variables) to the current geographical distribution of species in terms of presence/absence to fit species' realized niches and predict the distribution of suitable habitats in space and time. Therefore, SDMs can prove useful in conservation by helping managers to visualize the invasive potential of IAPs (Guisan & Thuiller, 2005) in AHCV. For instance, Thalmann et al. (2014) estimated the current and future susceptibility of PAs to 9 IAPs in Georgia with SDMs. However, as human‐mediated biological invasions are recent phenomena presenting an ongoing expansion, there is a need to include a broader number of potential IAPs (e.g., IAPs not occurring yet in the region of interest) for the prevention of biological invasion.

Situated in the Caucasus mountain ranges, Georgia is known for its high plant biodiversity and endemism. The flora of Georgia encompasses about 4,400 native species of vascular plants and 380 non‐native plant species, from which 16 are classified as invasive (Kikodze et al., 2010). Approximately 21% of the Georgian flora is endemic to the Caucasus region, 278 of them being strictly endemic to Georgia (Gagnidze et al., 2002; Solomon et al., 2014). This high biodiversity is recognized by the national protection of 43 areas in Georgia, which follow the IUCN guidelines (Dudley, 2008) and are general indicators of high biodiversity. While PAs are in general indicators of high biodiversity, those areas are not necessarily encompassing high plant endemism or endangered species (i.e., critically endangered, endangered, and vulnerable following IUCN guidelines; Dudley, 2008), as it was shown for Armenia (Fayvush, Tamanyan, Kalashyan, & Vitek, 2013). Fayvush et al. (2013) showed that the protected natural areas of Armenia are missing important hotspots of plant endemism and even fail to preserve adequately half of the plants under categories Critically endangered (CR), endangered (EN), and vulnerable (VU) by IUCN classification. Countries with high biodiversity and a high number of strictly endemic and subendemic plant species, such as Georgia (Gagnidze et al., 2002; Solomon et al., 2014), have an international responsibility to protect them adequately (see Convention on Biological Diversity: https://www.cbd.int/convention/). Due to the high conservation value of endemic species and their susceptibility to IAPs and climate changes (e.g., Li et al., 2013; Barrett & Yates, 2015; Urban, 2015; Zhang et al., 2016), areas of high plant endemism should be considered for the assessment of future impacts on endemic plant species in AHCV. In addition, reconciling potential inconsistencies between the distribution of PAs and areas of high endemic/endangered species richness would lead to a better prediction of AHCV and conflict zones between IAPs and AHCV.

In this study, we identified AHCV in Georgia by combining the delimitation of the PAs with areas of high plant endemism. Here, we consider AHCV as regions exhibiting exceptional botanical species richness and/or particular assemblage of rare, and threatened and/or endemic plant species. We then estimated the susceptibility of AHCV to invasion by 27 IAPs, including IAPs that still have not invaded Georgia and have the potential to become invasive, using SDMs for present and future climate conditions. More specifically, we investigate the following questions: (1) Are Georgia's Protected Areas representative of areas of high plant endemism? (2) Which areas and AHCV in Georgia are more susceptible to IAPs and how will this susceptibility change under future climate scenarios?

## 2 MATERIAL AND METHODS

### 2.1 Study area

**Figure 1:**
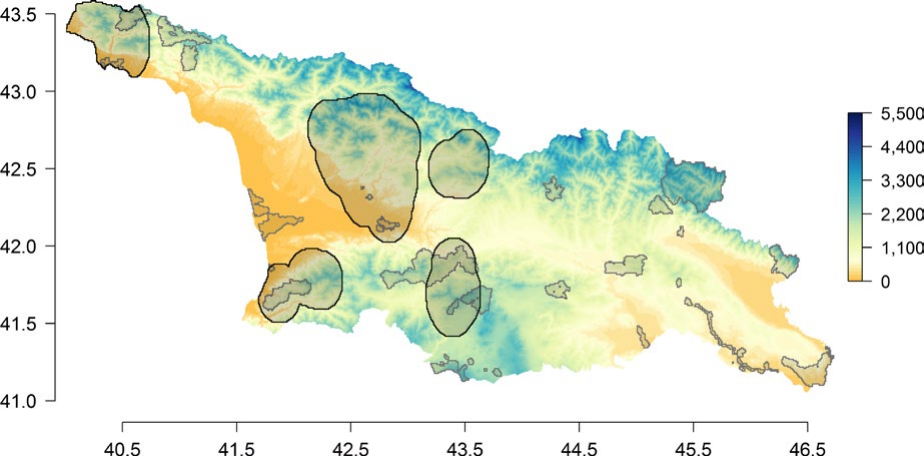
Map of the study area representing the Georgian country with its elevation range (meter). The colored scale represents the elevation gradient. The protected areas are shown as gray‐shaded frames and areas of high plant endemism as black‐rimmed frames

The study area corresponds to the boundary of the Georgian country situated in the Caucasus region and covers an area of approximately 69,700 km^2^ (Figure 1). Georgia is situated between two important mountain ranges, the Greater Caucasus range (north part culminating at 5,500 m) and the Lesser Caucasus range (South part). This particular topography protects Georgia from colder air masses from the north and partially from the hot and dry air masses from the south (Lydolph, 1977). Georgia displays diverse climate and vegetation types, ranging from subtropical in the western part, to continental in the eastern part, and temperate at high elevation.

### 2.2 Areas of high conservation value

We identified AHCV based on Georgia's Protected Areas and the areas of high plant endemism. Protected areas in Georgia follow the IUCN guidelines with categories ranging from “Strict Nature Reserve” (Category Ia) to “Protected Area with sustainable use of natural resources” (Category VI; Dudley, 2008). We excluded natural monuments (i.e., Category III) because of their small size and their priority on protecting natural features rather than the ecosystem (Dudley, 2008). The total surface of the 39 selected PAs represents approximately 7% of Georgia's total area.

Out of 278 endemic species occurring in Georgia, we retained 114 plant species for which we found occurrence data in Georgia (*n* = 765, Figure S1). From the selected endemic species, 88 species are strictly endemic to Georgia and 26 species are Caucasian endemics (Table S1 and S2). Occurrence data were collected from the online database Global Biodiversity Information Facility (GBIF; http://www.gbif.org/) and from rich herbarium collections of the National Herbarium of Georgia (TBI), the herbarium of National Museum of Georgia (TGM), the Ivane Javakhishvili Tbilisi State University Herbarium, and the Batumi Botanical Garden (BAT) In addition, all volumes of the second edition of Flora of Georgia were consulted (except the large family Poaceae that is still not covered in the already published sources), which include information on the localities where the species was collected, frequently on the landmark level but always indicating the nearest settlement/urban areas. We verified that our subset of 114 species was a phylogenetically nonbiased sample of all the endemics. To do so, we constructed the phylogenetic tree of all Georgian endemics (*n* = 279) and the selected georeferenced endemics (*n* = 114) with the online program Phylomatic (Webb & Donoghue, 2008) and calculated the branch lengths for both trees with the program Phylocom Version 4.2 (Webb et al., 2005). We then calculated the mean pairwise distance (mpd) of the selected 114 endemic plants with the “picante” package (Blomberg, Garland & Ives, 2003; Kembel et al., 2010) in R (R Development Core Team, 2014, Version 3.1.2) and compared it to a null distribution generated from 1,000 random sampling of 114 species across the tips of the phylogenetic tree. We checked whether the mpd value is similar to random samples of the total species pool with a two‐tailed test. The mpd value of the 114 georeferenced endemics showed no significant difference to the mpd values of the 114 randomly sampled endemics (two‐tailed test: *p*‐value: .372), indicating that our subset of 114 species is a phylogenetically nonbiased sample of all the Georgian endemics.

From endemic occurrences, we generated a map of endemic species richness per pixel at a spatial resolution of 0.0083° (~1 km), using a focal moving window of 0.5° across the Georgian landscape and using a smoothing procedure with the “focal” function of the R package “raster” (Hijmans & Van Etten, 2014) as the mean of the values in a 0.083° neighborhood (i.e., 10 pixels radius). We considered pixels with more than 15 endemics as areas of high plant endemism and buffered this area by 0.5° in order to take into account the variability introduced by the focal window in the mapping procedure. The threshold of 15 species was chosen to insure that the selected areas of high plant endemism cover approximately 20% of Georgia's surface (see Figure S2 for a cover representing approximately 10%, 5%, and 1% of Georgia). We considered this area as area of high plant endemism, representing 20.8% of Georgia's surface (Figure 1). According to the worldbank database on protected terrestrial areas (https://data.worldbank.org/indicator/), which ranks countries according to their total surface protected, Georgia is only ranked 139 on 209 countries (i.e., 8.4% of its surface protected). This very low amount of PAs contrasts with the 71 countries having more than 20% of their surface protected, as well as with the neighboring country of Armenia situated in the Lesser Caucasus mountain range with 24.8% of its surface protected. Considering an endemic area covering approximately 20% of the Georgian country ensures to reach similar surfaces for AHCV compared to other worldwide or neighboring countries. As observations of endemics are rare, we used the previous focal window approach, buffers and threshold to lower the impact of the low data availability and to obtain a regional estimate of the endemic richness. Using a smaller focal window, buffers and thresholds would lead to increase the chance of missing areas of high plant endemism due to lower data availability at a local scale. The selected endemic areas represent the Georgian potential areas of high plant endemism based on georeferenced observations. We are not aware of any published sources on areas of concentration of endemic species, which would allow to validate our selected areas of high plant endemism or to assess if some them are missing in some areas of Georgia. Finally, we defined AHCV in Georgia as the combination of areas of high plant endemism and Georgia's protected areas, together representing 27.8% of the Georgian surface (Figure 1).

### 2.3 Invasive alien plants

The non‐native flora of Georgia comprises 380 species (excluding cultivated species which are not, or only rarely found in the natural environment), representing 8.9% of the total flora of Georgia, of which 16 plant species are actually considered as major threat and classified as IAPs (Kikodze et al., 2010; Richardson et al., 2000). We selected 27 IAPs (including all 16 recognized IAPs in Georgia) presenting more than 100 occurrences worldwide and showing high potential to become invasive based on their status in other European countries with similar climatic conditions (see Table S3 for details on the selected IAPs). Among the selected IAPs, the most noxious ones are *Ambrosia artemisiifolia*,* Robinia pseudoacacia*, and *Ailanthus altissima* (see Thalmann et al., 2014). Occurrence data (*N* = 374,232) were collected from several Georgian herbaria (i.e., Georgian State Museum, Tbilisi State University Herbarium, and Batumi Botanical Herbarium), from occurrence points collected in the field with a GPS device (Garmin GPSMAP 64), from a previous study (Thalmann et al., 2014) and from the online database GBIF (http://www.gbif.org/; see Figure S3). GBIF data are known to be subject to spatial bias and accuracy issues, especially in species identification, taxonomy, or GPS precision (e.g., Beck et al., 2014; Meyer et al., 2016) . Thus, all IAPs used in this study were checked for synonyms in the data collection, missing or clearly false locality coordinates were removed from the analyses, and our modeling procedure includes a disaggregation strategy reducing the potential spatial bias (Beck et al., 2014). Because 28% of the downloaded GBIF data contained no information about the precision of the observation, we decided to keep all the observations. So far, IAP occurrences in Georgia are mainly found in the Adjara region in southwestern Georgia and in the Samegrelo region in western Georgia and around the cities of Kutaisi and Tbilisi (see Figure S4). However, those distributions may be the result of sampling bias close to cities and along main roads.

### 2.4 Environmental predictor variables

To model the potential distribution of IAPs, we used six climatic variables known to have a strong influence on plant physiological performance and survival and used by a previous study on IAPs in Georgia (Bartlein et al., 1986; Thalmann et al., 2014). We used maximal temperature of the warmest month (tmax), minimal temperature of the coldest month (tmin), temperature annual range (tar), mean temperature of the wettest month (twetq), precipitation of wettest month (pwet), and precipitation of the driest month (pdry). Environmental variables were obtained from the WorldClim database (Hijmans et al., 2005) with a spatial resolution of 0.0083° (~1 km). We extracted the values of these climatic predictors for all IAPs observations worldwide (*n* = 374,232) and checked for multicollinearity with pairwise correlations in order to avoid spurious model calibrations (Guisan & Thuiller, 2005). It is common practice (e.g., Aguirre‐Gutiérrez et al., 2013) to retain only predictors with pairwise correlation < |.7|. As the Pearson correlations were globally lower than this threshold except for tar and tmin (*r* = −.74), we kept all predictor variables as used in the study of Thalmann et al. (2014).

### 2.5 Species distribution models

To determine the potential distribution of the 27 selected IAPs in Georgia, we built SDMs by relating occurrence observations to the six environmental variables using custom code in R. Models have the tendency to vary among the different statistical techniques (Elith et al., 2006; Thuiller et al., 2004). Thus, we ran an Ensemble approach (Araújo & New, 2007) by averaging the results of five commonly used statistical techniques: generalized linear model (McCullagh & Nelder, 1989), gradient boosting model (Friedman, 2001; Ridgeway, 1999), general additive models (Guisan & Zimmermann, 2000), Random Forest (Breiman, 2001), and maximum entropy (Phillips et al., 2006; Elith et al., 2011). Models were calibrated with worldwide occurrences (i.e., native and invaded ranges) and projected in Georgia at a spatial resolution of 0.0083° (~1 km^2^). We chose to include all the occurrence data available for the native and invaded range in order to account for possible niche shifts in the invaded area (even if they are rare among species; see Petitpierre et al., 2012) and to obtain more accurate models for predicting the potential distribution of the species in the invaded range (Beaumont et al., 2009; Broennimann & Guisan, 2008).

To calibrate the models, we firstly avoided spatial autocorrelation in the presences data using the disaggregation tool provided by the “ecospat” package (Broennimann et al., 2017) in R by setting a minimal distance of 0.083° between presences. We then selected randomly a set of pseudo‐absences (also known as background data; Wisz & Guisan, 2009). Pseudo‐absences were drawn within the biomes occupied by the species. For this task, we used 14 biomes from the Terrestrial Ecoregions of the World (Olson et al., 2001). With this approach, we ensure that pseudo‐absences are drawn only from regions where the species occurs and that have been accessible as the species speciated (Barve et al., 2011; Thalmann et al., 2014) . We selected randomly the same amount of pseudo‐absences than presences, gave equal weights to presences and pseudo‐absences in the calibration of the model, and averaged several runs (Barbet‐Massin et al., 2012). We ran 10 iterations of models, avoiding spatial autocorrelation among presences, and sampling each time the pseudo‐absences within the biomes where the species occurs.

To evaluate the capacity of the models to correctly predict the presence and absence of the species at the global scale, we used the previously selected set of presences and pseudo‐absences for each iteration. Models were calibrated on a random sample of 70% of the presences and pseudo‐absences data, and evaluated on the remaining 30%. We used the area under the ROC plot curve (AUC; Fielding & Bell, 1997) and the True Skill Statistics (TSS; Allouche et al., 2006), which both evaluate the ability of the model to discriminate presences from absences. AUC varies between 0 (counter prediction) and 1 (perfect prediction), 0.5 meaning random predictions. TSS is scaled between −1 and 1, 0 meaning random predictions. As biological invasions are ongoing processes, the selected absences may not be representative of true absences and may thus bias the evaluation (Václavík & Meentemeyer, 2009). We thus added one presence‐only evaluator: the sensitivity calculated on predictions binarised with the threshold providing the best TSS. The sensitivity is a threshold‐dependent evaluator corresponding to the rate of presences correctly classified by the model. Models are considered to have reliable prediction performances with AUC values > 0.70 (i.e., excellent AUC  >  0.90; good 0.80  <  AUC  <  0.90; fair 0.70  <  AUC  <  0.80; poor AUC  <  0.70; see Swets, 1988) and TSS values > 0.40 (i.e., excellent TSS  >  0.75; good 0.40  <  TSS  <  0.75; poor TSS  <  0.40; see Landis & Koch, 1977). Finally, evaluators were averaged for all models and replicates (10 iterations).

In addition, we evaluated the predictive performance of the models in Georgia by calibrating the models with all occurrences and pseudo‐absences present outside Georgia (i.e., presences and pseudo‐absences inside Georgia were removed from the data) and by evaluating the models on presences and random pseudo‐absences selected in Georgia by following the same procedure as above for pseudo‐absences selection. Predictive performance of the models in Georgia was only assessed for 19 IAPs species that presented more than 10 occurrences after avoiding spatial autocorrelation. Evaluators were averaged for all models and replicates (10 iterations).

**Figure 2:**
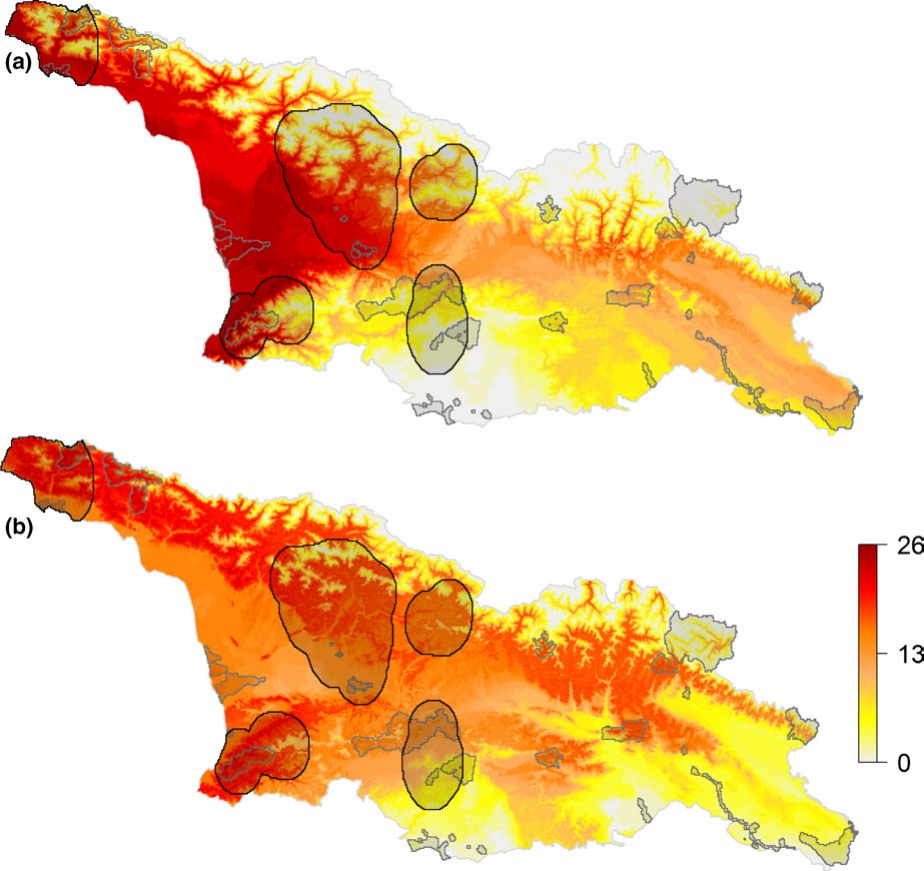
Invasive alien plant species richness in Georgia for the (a) present climate and (b) future climate for the year 2050 (RCP 8.5 IPSL‐CM5A‐LR climate change scenario). The colored scale represents the species richness. Each pixel represents the invasive alien plant richness on this site location (resolution: 1 km^2^). The protected areas are shown as gray‐shaded frames and areas of high plant endemism as black‐rimmed frames

Finally, we averaged the 10 projections of the five algorithms together in a single map and binarised it using a threshold maximizing the TSS. This threshold was obtained by averaging the threshold estimated for each algorithm and iterations with the “optimal.thresholds” function from the “PresenceAbsence” package (Freeman & Moisen, 2008) in R. All binarised maps were then combined into one consensus map representing the species richness of IAPs, used in this study as a metric of invasion susceptibility (Figure 2a). We finally summed species mean predictions of habitat suitability (mean of the five different algorithms) of all IAPs together in a single map representing a global invasion risk and found a strong and significant correlation between this map and the richness map inferred from binary conversion (Spearman correlation: *r* > .97, *p*‐value < .001; see Figures S5 and S6). For the following analyses, we only used species richness of IAPs as a metric of invasion susceptibility.

The choice of the selected pseudo‐absences sampling strategy can profoundly affect SDM predictions (e.g., Gu & Swihart, 2004), which may vary between statistical models (Barbet‐Massin et al., 2012). To assess the reliability of our results, we compared our IAPs richness map to richness maps obtained with two different pseudo‐absences sampling strategies: (1) a larger number of randomly selected pseudo‐absences (here 5k more than number of occurrences) with equal weighting for presences and pseudo‐absences as recommended by Barbet‐Massin et al. (2012) and (2) a biased background using a target‐group sampling approach where a same number of pseudo‐absences and occurrences are randomly selected from the total IAPs occurrences (see Merow et al., 2013; Phillips et al., 2009). All pseudo‐absences were selected into the biomes where the species occurs. Overall, we found a very strong correlation between our initial IAPs richness map and the richness maps derived from higher number of pseudo‐absences (Spearman correlation: *r* = .98, *p*‐value < .001) and from target‐group pseudo‐absences selection (Spearman correlation: *r* = .91, *p*‐value < .001), which supports and gives confidence to our methodology.

### 2.6 Climate change scenarios

To model the future distribution of invasive plant species, we used two different climate projection scenarios for 2050 (i.e., for the 2041–2060 time period) with two different global climate models (GCMs). We used two representative concentration pathway scenarios: RCP 4.5 (moderate, mean global warming increase of 1.4°C) and RCP 8.5 (more severe, mean global warming increase of 2.0°C). We used the GCMs of the Fifth Assessment IPCC report (AR5, 2014); HadGEM2‐AO (Martin et al., 2011) and IPSL‐CM5A‐LR (Dufresne et al., 2013). All variables were obtained from the WorldClim database (Hijmans et al., 2005) with a spatial resolution of 0.0083° (~1 km^2^). We projected all models for the invasive plant species with the two different scenarios assuming unlimited dispersal of species, which has been shown to be a close approximation to using dispersal kernels in mountains (Engler et al., 2009).

### 2.7 Species richness of IAPs as a metric for invasion risk

We evaluated present and future invasion risks by IAPs for the entire territory of Georgia and for AHCV using predicted IAP richness as a metric of invasion susceptibility and by comparing present to future changes in four richness classes with the same ranges (number of IAPs predicted: 0, i.e., absent; 1–9, i.e., low; 10–18, i.e., medium; and 19–26, i.e. high). Then, for each species in Georgia and AHCV, we calculated a potential range filling index (PRF) and an actual range filling index (ARF) following a procedure modified from Descombes et al. (2016). PRF is defined as the percent of area predicted to be suitable in the total area available (predicted area of the species in Georgia/AHCV divided by the total area of Georgia/AHCV). In order to estimate the actual range occupied by the species in Georgia/AHCV, we buffered current occurrences of IAPs by 10 km. ARF is defined as the percent of area actually occupied by the species in the predicted potential distribution of the species in Georgia/AHCV (actual estimated area of the species in Georgia/AHCV divided by the predicted area of the species in Georgia/AHCV). Note that ARF represents the minimal known distribution of the IAPs and that it could be underestimated due to sampling issues.

## 3 RESULTS

### 3.1 AHCV in Georgia

Our analysis showed that large parts of the areas of high plant endemism are located in central Georgia, north of Kutaisi in the regions of Racha, Imereti, and Samtskhe‐Javakheti and in the northwestern part in Abkhazia (Figure 1). We found that only 9.4% of areas of high plant endemism are inside PAs, showing a clear lack of protection for endemic plant species in Georgia (Figure 1).

### 3.2 Models performance

Distribution models calibrated and evaluated at the global scale showed reliable predictions for the 27 selected IAPs, with good to excellent AUC values (0.849 ≤ AUC ≤ 0.981), good to excellent TSS values (0.572 ≤ TSS ≤ 0.902), and good sensitivity values (0.776 ≤ sensitivity ≤ 0.977; Table S4). Overall, evaluations of distribution models calibrated at the global scale and evaluated in Georgia for 19 IAPs showed reliable predictions with fair to excellent AUC values (0.704 ≤ AUC ≤ 0.941), good to excellent TSS values (0.497 ≤ TSS ≤ 0.849), and good sensitivity values (0.815 ≤ sensitivity ≤ 0.982 (Table S5; Figure S7), except for *A. artemisiifolia*,* Chenopodium album*,* Conyza canadensis*,* Elsholtzia ciliata*,* Galinsoga parviflora*,* R. pseudoacacia*, and *Solidago canadensis* (Table S5). Differences between global and Georgian evaluations for AUC and TSS evaluators indicate that *A. artemisiifolia*,* C. album*,* C. canadensis*,* E. ciliata*,* G. parviflora*,* R. pseudoacacia*, and *S. canadensis* are probably still not at equilibrium in Georgia, resulting in weak predictive abilities when models are evaluated with Georgian occurrences only (Table S5). However, the evaluation of distribution models when using a presence‐only evaluator (i.e., sensitivity) shows that actual occurrences in Georgia are well predicted by the distribution models, with sensitivity values >= 0.775 for most of the IAPs (Table S5; Figure S7), except for *E. ciliata* (mean ± *SD*; sensitivity = 0.597 ± 0.212) and *G. parviflora* (sensitivity = 0.636 ± 0.170).

### 3.3 Current and future distribution of IAPs in Georgia

For the current climate, the SDMs predicted a high suitability for almost all selected 27 IAPs along the coastline in western Georgia and in central Georgia at lower altitudes (Figure 2a). Another area of high suitability for IAPs is in eastern Georgia north of Tbilisi, with up to 15 IAPs (Figure 2a). Approximately 11.4% of total Georgian area is not suitable for the IAPs investigated in this study and is mostly located at high elevation (Table 1, Figure 2a). The area suitable also decreased with the number of IAPs (Table 1). The potential distribution of IAPs in Georgia (i.e., PRF) varies highly between species (Table 2), ranging from 75% of Georgia' surface (*A. artemisiifolia*) to 0% (*Hydrocotyle vulgaris*; Table 2). However, the ARF of IAPs in the predicted potential distribution (i.e., PRF) is overall low, ranging from 31.6% (*Ulex europaeus*) to 0% (*H. vulgaris*), indicating that most suitable areas for IAPs are not invaded yet (Table 2).

**Table 1:** Threat potential of the 27 invasive alien plants (IAPs) for the present and the future in Georgia, potential area of high plant endemism (AHPE), protected areas (PAs), and area of high conservation values (AHCV; i.e., AHPE and PAs). Values correspond to the percent of predicted surface occupied by the different ranges of invasive species richness (0, 1–9, 10–18, and 19–26 species). Future predictions are for the RCP 8.5 IPSL‐CM5A‐LR climate change scenario for the year 2050 (see Table S6 for the results of the other future climate change scenarios)

IAPs richness	Georgia (%)		AHPE (%)		PAs (%)		AHCV (%)	
	Current	Future	Current	Future	Current	Future	Current	Future
0	11.4	2.2	4.4	0.1	21.6	6.1	9.9	2.0
1–9	39.5	33.6	33.9	10.4	44.3	36.9	36.2	18.4
10–18	29.0	53.2	28.9	53.6	20.8	39.6	26.6	49.4
19–26	20.1	11.0	32.8	36.0	13.3	17.4	27.3	30.3

**Table 2:** Potential and actual range filling of the 27 invasive alien plants (IAPs) for the present in Georgia and area of high conservation values (AHCV). Potential range filling (PRF) corresponds to the area of Georgia or AHCV (in %) predicted to be suitable for the species with our models. Actual range filling (ARF) corresponds to the area actually occupied by the species in the predicted potential distribution of the species (see Section “2” for details on the PRF and ARF calculations). Note that ARF represents the minimal known distribution of the IAPs and that it could be underestimated due to sampling issues

Species	Georgia		AHCV	
	PRF	ARF	PRF	ARF
*Ailanthus altissima*	47.3	15.3	44.0	13.7
*Ambrosia artemisiifolia*	75.0	22.0	76.0	21.3
*Amorpha fruticosa*	49.6	6.1	43.5	2.0
*Buddleja davidii*	19.3	8.0	24.8	8.7
*Chenopodium album*	68.8	18.3	85.4	17.7
*Clerodendrum bungei*	30.2	6.1	37.2	10.6
*Commelina communis*	50.3	17.0	57.4	28.8
*Conyza canadensis*	67.1	5.8	70.3	12.1
*Conyza graminifolia*	16.8	14.1	24.6	15.4
*Crassocephalum crepidioides*	10.8	14.3	5.8	31.8
*Elsholtzia ciliata*	74.5	6.0	76.4	3.7
*Galinsoga parviflora*	23.9	10.6	27.9	18.4
*Gleditsia triacanthos*	45.8	6.1	37.7	9.4
*Hydrocotyle vulgaris*	0.0	0.0	0.1	0.0
*Ixeridium dentatum*	13.1	24.6	15.4	25.6
*Miscanthus sinensis*	41.3	1.4	58.2	2.9
*Paspalum dilatatum*	27.9	12.1	26.3	24.3
*Paulownia tomentosa*	44.5	4.6	43.8	8.8
*Perilla nankinensis*	33.9	20.0	45.0	25.2
*Phytolacca americana*	60.1	16.9	55.3	32.1
*Polygonum thunbergii*	33.9	9.9	48.4	10.7
*Pueraria lobata*	22.8	2.5	26.3	6.0
*Robinia pseudoacacia*	53.6	17.4	53.7	13.4
*Solidago canadensis*	37.5	4.5	54.5	7.1
*Spiraea japonica*	60.3	1.4	75.7	3.0
*Ulex europaeus*	7.4	31.6	11.0	41.7
*Vitex rotundifolia*	16.8	3.6	19.4	6.4

Under future climate projections (i.e., year 2050), the suitability ranges for IAPs will shift toward higher altitudes in the higher and lesser Caucasus Mountains and toward the East of Georgia (Figures 2b and S8). Under the four climate change models, the areas with no suitability for IAPs will decrease compared to the present state (i.e., <7.6% of Georgia's surface; Table 1 and S6, Figures 2b and S8), resulting from an increase in suitability at high elevation sites. Overall, future climate projections show that there will be a global decrease in suitability for 1–9 and 19–26 IAPs (Tables 1 and S6, Figures 2b and S8) and suitability areas for 10–18 IAPs will drastically increase from 29% under current climate conditions up to 53.2% (Tables 1 and S6, Figures 2b and S8).

### 3.4 AHCVs at risk by IAPs

For the current climate, the AHCV most at risk due to predicted high suitability of IAPs is in central Georgia north of Kutaisi, in northwestern Georgia in Abkhazia, in western Georgia along the coastline in Samegrelo, and in southwestern Georgia in Adjara (Figure 2a). The areas with no suitability for IAPs in AHCV represent 9.9% of total AHCV area, while areas with suitability for 1–9, 10–18, and 19–26 IAPs represent 36.2%, 26.6%, and 27.3%, respectively (Table 1). The AHCV that is not or only barely affected by IAPs is the PAs in the far south in the lesser Caucasus Mountains and in the northeast in the higher Caucasus Mountains (Figure 2a). The potential distribution of IAPs in AHCV varies highly between species (Table 2), ranging from 76.4% of AHCV surface (*E. ciliata*) to 0.1% (*H. vulgaris*; Table 2). However, the ARF in AHCV is globally low, ranging from 41.7% (*U. europaeus*) to 0.1% (*H. vulgaris*), indicating that most suitable areas for IAPs are not invaded yet (Table 2).

Under the four future climate change models, the areas suitable for IAPs within AHCV will increase compared to the present state (i.e., <5.7% of AHCV surface; Tables 1 and S6, Figures 2b and S8). Overall, future climate projections in AHCV show that there will be a global decrease in suitability for 1–9 IAPs (Tables 1 and S6, Figures 2b and S8), suitability areas for 10–18 IAPs will drastically increase from 26.6% under current climate conditions up to 59.3% (Tables 1 and S6, Figures 2b and S8), and suitability areas for 19–26 IAPs will increase or decrease depending on the scenario (Tables 1 and S6, Figures 2b and S8).

Protected Areas will be slightly less affected by a predicted high suitability of IAPs compared to the total Georgia or AHCV (Table 1). Only the southern‐most PAs will stay unaffected and have a lower invasion susceptibility in the future. All other PAs will face a higher risk with increasing areas of high suitability for IAPs, notably in higher elevations. Interestingly, actual PAs seems to be located in areas with low suitability for IAPs (i.e., 21.6% coverage with no suitability for IAPs), while, in contrast, areas of high plant endemism are located on areas with very high IAPs richness (Table 1).

## 4 DISCUSSION

For the first time, we used an approach which includes both PAs and areas of high plant endemism to identify AHCV at the scale of a country. We found in the case of Georgia that PAs only cover a small portion of the areas of high plant endemism (i.e., 9.4%). We show that areas of high plant endemism are located in areas with higher suitability for IAPs compared to PAs, subjecting endemic plant species to high threats from IAPs. As areas of high plant endemism are located outside official Georgian Protected Areas, they have a lower chance to be included in IAP management programs.

### 4.1 Areas of high conservation value

Our finding reveals that using PAs as the only proxy for delimiting AHCV (e.g., Thalmann et al., 2014) may be of limited relevance. Protected Areas represent only 7% of the surface of Georgia, which translate the fact that resources allocated to conservation are limited to only a small fraction of areas with conservation value in order to maximize biodiversity protection in complementarity with other PAs in the network of PAs (Foxcroft et al., 2013). Using data on endemic species allows a more inclusive coverage of AHCV, with a surface reaching 27.4% of the surface of Georgia. Overall, we show that endemics are in large part distributed outside of PAs and are therefore at risk of competition by IAPs, but also by nonsustainable land use such as overgrazing, farming, or urbanization (Reidsma et al., 2006; Seto et al., 2012). A similar pattern was also found in Armenia (Fayvush et al., 2013), where protected natural areas are missing important hotspots of plant endemism and fail to preserve adequately half of the threatened plants according to the IUCN classification (i.e., critically endangered, endangered, and vulnerable). This is a strong signal that the already existing PAs in Georgia are not sufficient to protect Georgia's rich biodiversity.

The paucity of data on rare and endemic species is a common problem in conservation biology (Engler et al., 2004). Our data make no exception as it contains enough and sufficiently precise data for only 41% of the total number of Georgian endemic species. However, our subset of species is a phylogenetically nonbiased sample representative of Georgian endemic plant species. In addition, the area covered by our AHCV is more or less representative of biodiversity and endemic‐rich areas in Georgia, especially in Abkhazia and Ajara (in the northwesternmost and southwesternmost parts, respectively) supporting Tertiary flora and being known as refugia of remote geological epochs (e.g., Kolakowsky, 1961; Nakhutsrishvili, 2012). However, more efforts should be invested into recording endemic species to better define endemic‐rich areas in Georgia (i.e., especially in areas poorly investigated) using scientific‐based sampling strategies (e.g., random stratified sampling). Note that while reviewing the list of Georgian endemic plant species, we found out that 11 of them were not endemic, but were synonyms to species ranging beyond the Caucasus region. We thus strongly recommend to critically review and update the current list of Georgian endemic plant species and to put efforts into georeferencing the remaining endemics in order to better define endemic‐rich areas in Georgia.

### 4.2 Current and future AHCV at high risk by IAPs

Presently, the highest concentration of IAPs is in the Adjara region in southwest Georgia, around Tbilisi area, and in central Georgia around Kutaisi (Figure S4). The distribution of IAPs is mostly associated with human activity around urban areas (Hulme, 2003). In particular, high soil disturbance and increased traffic around big agglomerations may have further facilitated plant invasions (Catford et al., 2012), as well as garden escapes from botanical gardens (Hulme, 2015). Our results also demonstrate that all IAPs have largely not yet reached all their potential distribution in Georgia and AHCV (see PRF and ARF values; Table 2), testifying for the recent and ongoing expansion of biological invasions in Georgia. The dispersal of these species should be limited by implementing conservation actions, particularly to limit their dispersal in AHCV.

A rather alarming finding of our study is that suitable areas for IAPs will increase with warmer climate, notably toward higher altitudes in the greater and lesser Caucasus. Under future climate change scenarios, IAPs will disperse to higher elevation on newly available suitable areas, leading to a decrease in low IAPs richness areas at high elevation. On the other hand, we also observe a decrease in suitability to IAPs at low elevation (Figure 2), which means that climate change will also decrease the suitability of some IAPs in the western part of Georgia (Figure 2). However, the species' range response to climate change along elevation gradients is not expected to occur simultaneously with climate change, but with a species‐specific temporal lag explained by variation in physiological and demographic responses, altered biotic interactions and aspects of the physical environment (Alexander et al., 2018). Although plant communities at higher altitudes are expected to be at lower risk of invasion due to lower propagule pressure and stressful abiotic conditions (Petitpierre et al., 2016; Zefferman et al., 2015), with increased global connectivity and changing climate, the risk of plant invasions at high altitudes is also predicted to increase (Pauchard et al., 2009). Anticipating which areas are predicted to be at high risk of invasion should greatly increase the efficiency of prevention programs against IAPs (Pauchard et al., 2015).

### 4.3 From predictions of climatic suitability to local impact assessments

Our approach predicts the potential suitability for IAPs but cannot consider the true in situ impact of the IAPs on their environment. Our predictions of potential suitability could be improved using finer resolution SDMs (e.g., Descombes et al., 2016; Heinänen et al., 2012; Razgour et al., 2011) or by exploiting available knowledge at the species level. In a recent study, a scoring system for invasive species, based on their environmental and socio‐economic impact, was developed (Kumschick et al., 2015). The risk posed by these species could be refined using this scoring system. For example, for the same predicted suitability value, we can expect *R. pseudoacacia* (i.e., with a global environmental and socio‐economic impact score of 20) to have a 10‐fold higher in situ impact than *Phytolacca Americana* (global impact score of 2). In addition, trait‐based approaches could be used for assessing and mapping potential niche overlap between native and exotic species (Elleouet et al., 2014). However, those analyses need broad data on species morphological traits which are rare for our selected endemic plant species. Furthermore, our models, as every static distribution models based on presence‐only data, cannot predict at which stage of invasion the IAPs are in each region, even if some indicative indexes can be derived (see PRF and ARF values in this study; Descombes et al., 2016). The stage of invasion by IAPs, known by local experts, should be considered when selecting appropriate measures to take against these IAPs (Blackburn et al., 2011). Despite these limitations, knowing which AHCV in Georgia are currently colonized by IAPs and which AHCV show a high climatic suitability under present and future climate is an important information to initiate effective prevention measures against IAPs within AHCV. While AHCV with a high suitability for a large number of IAPs need the highest attention, the AHCV with a low number of highly suitable IAPs should not be ignored, as eradications are usually only successful if done at an early stage of invasion and as single IAP species can already have a huge impact on the whole ecosystem. Such “transformer species” include, for example, *A. artemisiifolia* and *R. pseudoacacia* (Ehrenfeld, 2010).

Recently, we established a monitoring program for IAPs at twelve monitoring sites in five different PAs in the west and in the east of Georgia, with the aim to follow the cover of both IAPs and members of the resident plant community in order to link the potential spread of IAPs with changes in plant biodiversity over time (Slodowicz et al., in press). The established monitoring scheme, presented at a workshop in Tbilisi in June 2015 to NGOs and national authorities, will also allow disentangling effects of biotic resistance (high diversity plots will be more resistance to IAP invasion) from direct negative effects on IAPs on plant diversity.

## 5 CONCLUSION

Our study showed that firstly, large AHCV with high plant endemism in Georgia are currently located outside of PAs and thus beyond the scope of established conservation management. Here, populations of rare and endemic plant species are specifically at risk due to IAPs. Secondly, large parts of the AHCV are already struggling with high numbers of IAPs, notably in Adjara and Samegrelo in southwestern Georgia, in central Georgia around Kutaisi and in eastern Georgia north of Tbilisi. Thirdly, many IAPs are predicted to shift with changing climate toward higher altitudes and toward the east, potentially threatening AHCV in south‐central Georgia.

Our study provides a good baseline for decision makers and stakeholders on where and how resources should be invested in the most efficient way to protect Georgia's high plant richness from IAPs (Maxwell et al., 2009). Various management tools are available ranging from programs to prevent the introduction of IAPs (e.g., Leung et al., 2002), eradication (Panetta et al., 2011), or mitigation through physical (cutting), chemical, or biocontrol programs (Müller‐Schärer & Collins, 2012; Müller‐Schärer & Schaffner, 2008).

## CONFLICT OF INTEREST

None declared.

## AUTHOR CONTRIBUTIONS

DS, HMS, OB, and DK involved in the study design; DS and PD involved in data interpretation; DS involved in drafting of the first manuscript; PD involved in running the SDMs; DS and DK involved in data collection; DS, PD, and OB involved in data analysis. HMS conceived the original idea and supervised the project; all involved in revising and final approval of the manuscript.
