# Supplementary material for: Areas of high conservation value at risk by plant invaders in Georgia under climate change
Source: Ecol Evol. 2018 Apr 2;8(9):4431–42. doi: 10.1002/ece3.4005 (PMC5938453; doi:10.1002/ece3.4005)

Research article for *Ecology and Evolution*

**Areas of high conservation value at risk by plant invaders in Georgia under climate change**

Daniel Slodowicz^a^*^+^, Patrice Descombes^b, c+^, David Kikodze^d^, Olivier Broenimann^e^, Heinz Müller-Schärer

^a^Department of Ecology and Evolution, University of Fribourg, Chemin du Musée 10, CH-1700 Fribourg, Switzerland

^b^Swiss Federal Research Institute WSL, Zürcherstrasse 111, CH-8903 Birmensdorf, Switzerland

^c^Landscape Ecology, Institute of Terrestrial Ecosystems, ETH Zürich, Universitätstrasse 16, CH-8092 Zürich, Switzerland

^d^Institute of Botany, Ilia State University, Botanikuri street 1, GE-0105 Tbilisi, Georgia

^e^Department of Ecology and Evolution, University of Lausanne, Biophore, CH-1015 Lausanne, Switzerland

*Corresponding author

^+^Co first authorship

**Supporting Information**

**Table S1** List of Georgian endemic plant species. Only the georeferenced ones were used for defining areas with high plant endemism.

| **Family** | **Scientific name** | **Georeferenced** | **Number of occurences** |
| --- | --- | --- | --- |
| Amaryllidaceae | *Allium albovianum* | no |  |
| Amaryllidaceae | *Allium chevsuricum* | yes | 1 |
| Amaryllidaceae | *Allium gracilescens* | no |  |
| Amaryllidaceae | *Allium otschiauriae* | no |  |
| Amaryllidaceae | *Galanthus kemulariae* | yes |  |
| Amaryllidaceae | *Galanthus ketzkhovelii* | yes | 8 |
| Amaryllidaceae | *Galanthus schaoricus* | no |  |
| Apiaceae | *Angelica adzharica* | no |  |
| Apiaceae | *Astrantia colchica* | no |  |
| Apiaceae | *Bupleurum abchasicum* | no |  |
| Apiaceae | *Carum grossheimii* | yes | 11 |
| Apiaceae | *Chymsydia colchica* | yes | 1 |
| Apiaceae | *Cnidium grossheimii* | no |  |
| Apiaceae | *Cnidium mandenovae* | yes | 1 |
| Apiaceae | *Cnidium pauciradiatum* | yes | 1 |
| Apiaceae | *Cryptotaenia flahaultii* | no |  |
| Apiaceae | *Heracleum egrissicum* | no |  |
| Apiaceae | *Heracleum grossheimii* | no |  |
| Apiaceae | *Heracleum ossethicum* | yes | 11 |
| Apiaceae | *Heracleum sommieri* | yes | 7 |
| Apiaceae | *Pimpinella schatilensis* | yes | 1 |
| Apiaceae | *Polylophium panjutinii* | yes | 1 |
| Asparagaceae | *Muscari alpanicum* | yes | 1 |
| Asparagaceae | *Ornithogalum imereticum* | yes | 1 |
| Asparagaceae | *Scilla otschiauriae* | no |  |
| Aspleniaceae | *Asplenium hermanii-christii* | yes | 2 |
| Asteraceae | *Achillea sedelmeyeriana* | yes | 2 |
| Asteraceae | *Anthemis emiliae* | yes | 3 |
| Asteraceae | *Anthemis saguramica* | no |  |
| Asteraceae | *Anthemis schischkiniana* | yes | 5 |
| Asteraceae | *Centaurea bagadensis* | no |  |
| Asteraceae | *Centaurea bella subsp. nathadzeae* | no |  |
| Asteraceae | *Centaurea transcaucasica subsp. georgica* | no |  |
| Asteraceae | *Cirsium albowianum* | no |  |
| Asteraceae | *Cirsium fominii* | no |  |
| Asteraceae | *Cirsium imereticum* | yes | 14 |
| Asteraceae | *Cirsium kemulariae* | no |  |
| Asteraceae | *Cirsium oblongifolium* | no |  |
| Asteraceae | *Cirsium sosnowskyi* | yes | 19 |
| Asteraceae | *Echinops foliosus* | no |  |
| Asteraceae | *Echinops galaticus subsp. ossicus* | no |  |
| Asteraceae | *Echinops sphaerocephalus subsp. cirsifolius* | no |  |
| Asteraceae | *Galatella eldarica* | no |  |
| Asteraceae | *Hieracium abakurae* | no |  |
| Asteraceae | *Hieracium brittatense* | no |  |
| Asteraceae | *Hieracium chromolepium* | no |  |
| Asteraceae | *Hieracium concinnidens* | no |  |
| Asteraceae | *Hieracium haematoglossum* | no |  |
| Asteraceae | *Hieracium laetevirens* | no |  |
| Asteraceae | *Hieracium macrolygodes* | no |  |
| Asteraceae | *Hieracium retroversilobatum* | no |  |
| Asteraceae | *Hieracium subsimplex* | no |  |
| Asteraceae | *Hieracium turfosum* | no |  |
| Asteraceae | *Jurinea exuberans* | no |  |
| Asteraceae | *Kemulariella tugana* | yes | 8 |
| Asteraceae | *Petasites georgicus* | yes | 6 |
| Asteraceae | *Podospermum grigoraschvilii* | no |  |
| Asteraceae | *Podospermum idae* | yes | 2 |
| Asteraceae | *Psephellus adjaricus* | yes | 1 |
| Asteraceae | *Psephellus kacheticus* | yes | 2 |
| Asteraceae | *Psephellus kolakovskii* | yes | 4 |
| Asteraceae | *Pyrethrum marionii* | no |  |
| Asteraceae | *Scorzonera ketzkhowelii* | yes | 1 |
| Asteraceae | *Scorzonera kozlowskyi* | no |  |
| Asteraceae | *Senecio similiflorus* | no |  |
| Asteraceae | *Solidago turfosa* | yes | 6 |
| Asteraceae | *Tragopogon colchicus* | yes | 4 |
| Asteraceae | *Tragopogon ketzkhovelii* | yes | 4 |
| Asteraceae | *Tragopogon makaschwilii* | yes | 1 |
| Asteraceae | *Tragopogon meskheticus* | yes | 2 |
| Asteraceae | *Tragopogon otschiaurii* | no |  |
| Asteraceae | *Tripleurospermum szowitzii* | yes | 16 |
| Berberidaceae | *Gymnospermium smirnovii* | yes | 2 |
| Betulaceae | *Betula megrelica* | no |  |
| Betulaceae | *Corylus abchasica* | no |  |
| Betulaceae | *Corylus colchica* | yes | 10 |
| Betulaceae | *Corylus egrissiensis* | no |  |
| Betulaceae | *Corylus imeretica* | no |  |
| Betulaceae | *Corylus kachetica* | yes | 6 |
| Boraginaceae | *Omphalodes kusnetzovii* | no |  |
| Boraginaceae | *Paracynoglossum imeretinum* | yes | 16 |
| Brassicaceae | *Arabis colchica* | yes | 4 |
| Brassicaceae | *Arabis kazbegi* | yes | 3 |
| Brassicaceae | *Barbarea ketzkhovelii* | yes | 4 |
| Brassicaceae | *Callothlaspi abchasicum* | yes | 1 |
| Brassicaceae | *Draba meskhetica* | yes | 1 |
| Brassicaceae | *Draba mingrelica* | yes | 7 |
| Brassicaceae | *Erysimum caucasicum* | yes | 21 |
| Brassicaceae | *Erysimum contractum* | no |  |
| Brassicaceae | *Erysimum subnivale* | yes | 1 |
| Campanulaceae | *Campanula armazica* | yes | 1 |
| Campanulaceae | *Campanula bzybica* | yes | 5 |
| Campanulaceae | *Campanula calcarea* | yes | 5 |
| Campanulaceae | *Campanula dzaaku* | no |  |
| Campanulaceae | *Campanula engurensis* | no |  |
| Campanulaceae | *Campanula fonderwisii* | no |  |
| Campanulaceae | *Campanula hieracioides* | no |  |
| Campanulaceae | *Campanula imeretina* | yes | 7 |
| Campanulaceae | *Campanula irinae* | yes | 2 |
| Campanulaceae | *Campanula jadvigae* | no |  |
| Campanulaceae | *Campanula kachetica* | no |  |
| Campanulaceae | *Campanula kantschavelii* | no |  |
| Campanulaceae | *Campanula kemulariae* | no |  |
| Campanulaceae | *Campanula kolakovskyi* | yes | 4 |
| Campanulaceae | *Campanula letschchumensis* | yes | 7 |
| Campanulaceae | *Campanula makaschvilii* | yes | 6 |
| Campanulaceae | *Campanula megrelica* | yes | 4 |
| Campanulaceae | *Campanula mirabilis* | no |  |
| Campanulaceae | *Campanula panjutinii* | yes | 1 |
| Campanulaceae | *Campanula paradoxa* | no |  |
| Campanulaceae | *Campanula radchensis* | yes |  |
| Campanulaceae | *Campanula raddeana* | no |  |
| Campanulaceae | *Campanula schistosa* | yes | 8 |
| Campanulaceae | *Campanula suanetica* | no |  |
| Campanulaceae | *Campanula symphytifolia* | no |  |
| Campanulaceae | *Symphyandra antiqua* | no |  |
| Caprifoliaceae | *Cephalaria sosnowskyi* | yes | 4 |
| Caprifoliaceae | *Scabiosa adzharica* | yes | 3 |
| Caprifoliaceae | *Scabiosa colchica* | yes | 18 |
| Caprifoliaceae | *Scabiosa imeretica* | yes | 10 |
| Caryophyllaceae | *Cerastium argenteum* | yes | 34 |
| Caryophyllaceae | *Cerastium svanicum* | no |  |
| Caryophyllaceae | *Dianthus abchasicus* | yes | 6 |
| Caryophyllaceae | *Dianthus azkurensis* | no |  |
| Caryophyllaceae | *Dianthus charadzeae* | yes | 6 |
| Caryophyllaceae | *Minuartia subuniflora* | yes | 8 |
| Caryophyllaceae | *Silene alexeji* | no |  |
| Caryophyllaceae | *Silene boissieri* | yes | 1 |
| Cistaceae | *Helianthemum georgicum* | no |  |
| Cornaceae | *Swida armasica* | no |  |
| Crassulaceae | *Sempervivum charadzeae* | no |  |
| Crassulaceae | *Sempervivum ermanicum* | no |  |
| Crassulaceae | *Sempervivum sosnowskyi* | yes | 2 |
| Cyperaceae | *Rhynchospora caucasica* | no |  |
| Fabaceae | *Astragalus argillosus* | no |  |
| Fabaceae | *Astragalus aspindzicus* | yes | 1 |
| Fabaceae | *Astragalus atenicus* | yes | 5 |
| Fabaceae | *Astragalus cyri* | yes | 1 |
| Fabaceae | *Astragalus doluchanovii* | no |  |
| Fabaceae | *Astragalus hirtulus* | yes | 9 |
| Fabaceae | *Astragalus kemulariae* | yes | 28 |
| Fabaceae | *Astragalus kozlovskyi* | yes | 1 |
| Fabaceae | *Astragalus leonidae* | yes | 4 |
| Fabaceae | *Astragalus magnificus* | no |  |
| Fabaceae | *Astragalus meskheticus* | no |  |
| Fabaceae | *Astragalus raddeanus* | yes | 24 |
| Fabaceae | *Astragalus schischkinii* | yes | 1 |
| Fabaceae | *Astragalus vardziae* | no |  |
| Fabaceae | *Cicer caucasicum* | yes | 1 |
| Fabaceae | *Genista adzharica* | no |  |
| Fabaceae | *Genista mingrelica* | no |  |
| Fabaceae | *Genista sachokiana* | no |  |
| Fabaceae | *Onobrychis angustifolia* | no |  |
| Fabaceae | *Onobrychis grossheimii* | no |  |
| Fabaceae | *Onobrychis kachetica* | yes | 13 |
| Fabaceae | *Onobrychis kemulariae* | no |  |
| Fabaceae | *Onobrychis meschetica* | yes | 23 |
| Fagaceae | *Quercus imeretina* | no |  |
| Gentianaceae | *Gentiana kolakovskyi* | no |  |
| Gentianaceae | *Gentiana rhodocalyx* | no |  |
| Hypericaceae | *Hypericum nordmannii* | no |  |
| Iridaceae | *Crocus autranii* | yes | 1 |
| Iridaceae | *Iris winogradowii* | no |  |
| Lamiaceae | *Galeopsis nana* | no |  |
| Lamiaceae | *Nepeta iberica* | no |  |
| Lamiaceae | *Salvia compar* | yes | 6 |
| Lamiaceae | *Satureja bzybica* | no |  |
| Lamiaceae | *Scutellaria helenae* | no |  |
| Lamiaceae | *Thymus ladjanuricus* | no |  |
| Lamiaceae | *Thymus sosnowskyi* | no |  |
| Lamiaceae | *Ziziphora borzhomica* | no |  |
| Lythraceae | *Trapa colchica* | yes | 1 |
| Lythraceae | *Trapa maleevii* | no |  |
| Malvaceae | *Alcea abchasica* | no |  |
| Malvaceae | *Alcea transcaucasica* | no |  |
| Malvaceae | *Hibiscus ponticus* | yes | 1 |
| Orobanchaceae | *Euphrasia adenocaulon* | yes | 2 |
| Orobanchaceae | *Euphrasia grossheimii* | no |  |
| Orobanchaceae | *Euphrasia kemulariae* | no |  |
| Orobanchaceae | *Euphrasia svanica* | no |  |
| Orobanchaceae | *Euphrasia woronowii* | no |  |
| Orobanchaceae | *Melampyrum alboffianum* | no |  |
| Orobanchaceae | *Orobanche quadrifida* | no |  |
| Orobanchaceae | *Pedicularis elisabethae* | no |  |
| Paeoniaceae | *Paeonia carthalinica* | yes | 2 |
| Paeoniaceae | *Paeonia lagodechiana* | yes | 3 |
| Paeoniaceae | *Paeonia majko* | no |  |
| Paeoniaceae | *Paeonia ruprechtiana* | no |  |
| Paeoniaceae | *Paeonia steveniana* | no |  |
| Papaveraceae | *Corydalis vittae* | no |  |
| Plantaginaceae | *Veronica colchica* | no |  |
| Plantaginaceae | *Veronica tumadzhanovii* | no |  |
| Poaceae | *Alopecurus longifolius* | no |  |
| Poaceae | *Bromopsis divaricata* | no |  |
| Poaceae | *Calamagrostis dmitrievae* | no |  |
| Poaceae | *Poa alexeenkoi* | no |  |
| Poaceae | *Triticum carthlicum* | no |  |
| Poaceae | *Triticum macha* | no |  |
| Poaceae | *Triticum palaeo-colchicum* | no |  |
| Poaceae | *Triticum timopheevi* | no |  |
| Poaceae | *Triticum zhukovskyi* | yes | 1 |
| Polygalaceae | *Polygala albowii* | no |  |
| Primulaceae | *Cyclamen colchicum* | no |  |
| Primulaceae | *Primula saguramica* | no |  |
| Ranunculaceae | *Aquilegia colchica* | no |  |
| Ranunculaceae | *Aquilegia gegica* | no |  |
| Ranunculaceae | *Delphinium elisabethae* | no |  |
| Ranunculaceae | *Delphinium ironorum* | no |  |
| Ranunculaceae | *Delphinium osseticum* | no |  |
| Ranunculaceae | *Delphinium thamarae* | no |  |
| Ranunculaceae | *Ficaria varia* | no |  |
| Ranunculaceae | *Pulsatilla georgica* | yes | 5 |
| Ranunculaceae | *Ranunculus migaricus* | no |  |
| Rhamnaceae | *Rhamnus cordata* | no |  |
| Rosaceae | *Alchemilla adelodictya* | no |  |
| Rosaceae | *Alchemilla alexandri* | no |  |
| Rosaceae | *Alchemilla aurata* | no |  |
| Rosaceae | *Alchemilla bakurianica* | no |  |
| Rosaceae | *Alchemilla capillacea* | no |  |
| Rosaceae | *Alchemilla cartalinica* | no |  |
| Rosaceae | *Alchemilla erectilis* | no |  |
| Rosaceae | *Alchemilla grandidens* | no |  |
| Rosaceae | *Alchemilla hypochlora* | no |  |
| Rosaceae | *Alchemilla hypotricha* | no |  |
| Rosaceae | *Alchemilla impolita* | no |  |
| Rosaceae | *Alchemilla indurata* | no |  |
| Rosaceae | *Alchemilla insignis* | no |  |
| Rosaceae | *Alchemilla microdictya* | no |  |
| Rosaceae | *Alchemilla obtegens* | no |  |
| Rosaceae | *Alchemilla pascualis* | no |  |
| Rosaceae | *Alchemilla subcrenatiformis* | no |  |
| Rosaceae | *Alchemilla suberectipila* | no |  |
| Rosaceae | *Alchemilla subsplendens* | no |  |
| Rosaceae | *Alchemilla woronowii* | no |  |
| Rosaceae | *Amygdalus georgica* | no |  |
| Rosaceae | *Potentilla imerethica* | no |  |
| Rosaceae | *Potentilla kemulariae* | no |  |
| Rosaceae | *Potentilla sommieri* | no |  |
| Rosaceae | *Potentilla sosnowskyi* | no |  |
| Rosaceae | *Rosa doluchanovii* | no |  |
| Rosaceae | *Rosa ermanica* | no |  |
| Rosaceae | *Rosa irysthonica* | no |  |
| Rosaceae | *Rosa transcaucasica* | no |  |
| Rosaceae | *Rubus abchaziensis* | no |  |
| Rosaceae | *Rubus adscharicus* | no |  |
| Rosaceae | *Rubus caucasigenus* | no |  |
| Rosaceae | *Rubus charadzeae* | no |  |
| Rosaceae | *Rubus cyri* | no |  |
| Rosaceae | *Rubus discernendus* | no |  |
| Rosaceae | *Rubus juzepczukii* | no |  |
| Rosaceae | *Rubus kachethicus* | no |  |
| Rosaceae | *Rubus ketzkhovelii* | no |  |
| Rosaceae | *Rubus kudagorensis* | no |  |
| Rosaceae | *Rubus lepidulus* | no |  |
| Rosaceae | *Rubus leptostemon* | no |  |
| Rosaceae | *Rubus longipetiolatus* | no |  |
| Rosaceae | *Rubus miszczenkoi* | no |  |
| Rosaceae | *Rubus moschus* | no |  |
| Rosaceae | *Rubus nakeralicus* | no |  |
| Rosaceae | *Rubus ochthodes* | no |  |
| Rosaceae | *Rubus ossicus* | no |  |
| Rosaceae | *Rubus platyphylloides* | no |  |
| Rosaceae | *Rubus ponticus* | no |  |
| Rosaceae | *Rubus woronowii* | no |  |
| Rubiaceae | *Galium praemontanum* | no |  |
| Santalaceae | *Thesium laxiflorum* | no |  |
| Sapindaceae | *Acer sosnowskyi* | no |  |
| Saxifragaceae | *Chrysosplenium albowianum* | no |  |
| Saxifragaceae | *Saxifraga abchasica* | no |  |
| Saxifragaceae | *Saxifraga kusnezowiana* | yes | 2 |
| Saxifragaceae | *Saxifraga trautvetteri* | no |  |
| Scrophulariaceae | *Scrophularia imerethica* | no |  |
| Scrophulariaceae | *Verbascum adzharicum* | no |  |
| Scrophulariaceae | *Verbascum sessiliflorum* | no |  |
| Tamaricaceae | *Reaumuria kuznetzovii* | yes | 2 |
| Ulmaceae | *Ulmus georgica* | no |  |
| Urticaceae | *Parietaria kemulariae* | no |  |
| Violaceae | *Viola orthoceras* | yes | 10 |

**Table S2** Georeferenced Caucasian endemic plant species, used for defining areas with high plant endemism, in addition to the Georgian endemics.

| **Family** | **Scientific name** | **Number of occurrences** |
| --- | --- | --- |
| Apiaceae | *Heracleum sphondylium subsp. cyclocarpum* | 12 |
| Apiaceae | *Laserpitium affine* | 3 |
| Apiaceae | *Peucedanum adae* | 6 |
| Apiaceae | *Seseli petraeum* | 6 |
| Apiaceae | *Seseli saxicolum* | 2 |
| Asteraceae | *Achillea latiloba* | 7 |
| Asteraceae | *Cirsium pugnax* | 16 |
| Asteraceae | *Kemulariella abchasica* | 8 |
| Asteraceae | *Kemulariella colchica* | 9 |
| Asteraceae | *Prenanthes abietina* | 17 |
| Asteraceae | *Psephellus abchasicus* | 8 |
| Asteraceae | *Psephellus carthalinicus* | 18 |
| Asteraceae | *Psephellus meskheticus* | 11 |
| Asteraceae | *Pyrethrum peucedanifolium* | 4 |
| Asteraceae | *Senecio pandurifolius* | 11 |
| Asteraceae | *Tanacetum punctatum* | 14 |
| Berberidaceae | *Epimedium colchicum* | 12 |
| Betulaceae | *Betula medwediewii* | 6 |
| Betulaceae | *Betula raddeana* | 11 |
| Boraginaceae | *Symphytum ibericum* | 15 |
| Brassicaceae | *Draba imeretica* | 9 |
| Campanulaceae | *Campanula dzyschrica* | 3 |
| Dryopteridaceae | *Dryopteris liliana* | 3 |
| Ericaceae | *Epigaea gaultherioides* | 4 |
| Ericaceae | *Rhododendron ungernii* | 10 |
| Fabaceae | *Genista abchasica* | 7 |

**Table S3** Selected 27 invasive alien plants.

| **Family** | **Scientific name** | **Status** | **Occurences worldwide** | **Occurences in Georgia** |
| --- | --- | --- | --- | --- |
| Simaroubaceae | *Ailanthus altissima* | invasive | 4301 | 47 |
| Asteraceae | *Ambrosia artemisiifolia* | invasive | 7879 | 120 |
| Fabaceae | *Amorpha fruticosa* | naturalized | 1432 | 9 |
| Scrophulariaceae | *Buddleja davidii* | subspontaneous | 20254 | 9 |
| Amaranthaceae | *Chenopodium album* | naturalized | 63157 | 88 |
| Verbenaceae | *Clerodendrum bungei* | invasive | 270 | 27 |
| Commelinaceae | *Commelina communis* | naturalized | 1173 | 76 |
| Asteraceae | *Conyza canadensis* | naturalized | 62350 | 51 |
| Asteraceae | *Symphyotrichum graminifolium* | unknown | 101 | 79 |
| Asteraceae | *Crassocephalum crepidioides* | invasive | 1223 | 23 |
| Lamiaceae | *Elsholtzia ciliata* | naturalized | 588 | 28 |
| Asteraceae | *Galinsoga parviflora* | naturalized | 14254 | 27 |
| Fabaceae | *Gleditsia triacanthos* | naturalized | 684 | 13 |
| Apiaceae | *Hydrocotyle vulgaris* | naturalized | 65376 | 12 |
| Asteraceae | *Ixeridium dentatum* | adventive | 800 | 18 |
| Poaceae | *Miscanthus sinensis* | invasive | 784 | 11 |
| Poaceae | *Paspalum dilatatum* | invasive | 6202 | 83 |
| Bignoniaceae | *Paulownia tomentosa* | subspontaneous | 943 | 10 |
| Lamiaceae | *Perilla nankinensis* | invasive | 709 | 51 |
| Phytolaccaceae | *Phytolacca americana* | naturalized | 2384 | 107 |
| Polygonaceae | *Polygonum thunbergii* | naturalized | 498 | 40 |
| Fabaceae | *Pueraria montana var. lobata* | naturalized | 843 | 9 |
| Fabaceae | *Robinia pseudoacacia* | invasive | 24916 | 54 |
| Asteraceae | *Solidago canadensis* | naturalized | 27052 | 13 |
| Rosaceae | *Spiraea japonica* | invasive | 645 | 11 |
| Fabaceae | *Ulex europaea* | adventive | 64780 | 36 |
| Verbenaceae | *Vitex rotundifolia* | invasive | 351 | 3 |

**Table S4** Evaluation of the species distribution models evaluated using world occurrences. The table shows the mean and standard deviation of AUC, TSS and Sensitivity evaluators (see methods for more details). All species show good evaluations.

|  | **AUC** | **TSS** | **SENSITIVITY** |
| --- | --- | --- | --- |
| *Ailanthus altissima* | 0.942 ± 0.014 | 0.770 ± 0.031 | 0.913 ± 0.027 |
| *Ambrosia artemisiifolia* | 0.944 ± 0.012 | 0.780 ± 0.028 | 0.915 ± 0.014 |
| *Amorpha fruticosa* | 0.899 ± 0.034 | 0.664 ± 0.069 | 0.853 ± 0.053 |
| *Buddleja davidii* | 0.981 ± 0.004 | 0.892 ± 0.010 | 0.948 ± 0.011 |
| *Chenopodium album* | 0.967 ± 0.007 | 0.823 ± 0.032 | 0.908 ± 0.025 |
| *Clerodendrum bungei* | 0.931 ± 0.028 | 0.798 ± 0.058 | 0.925 ± 0.041 |
| *Commelina communis* | 0.942 ± 0.024 | 0.803 ± 0.051 | 0.919 ± 0.031 |
| *Conyza canadensis* | 0.957 ± 0.010 | 0.776 ± 0.040 | 0.887 ± 0.040 |
| *Conyza graminifolia* | 0.849 ± 0.049 | 0.572 ± 0.090 | 0.776 ± 0.109 |
| *Crassocephalum crepidioides* | 0.882 ± 0.032 | 0.647 ± 0.053 | 0.810 ± 0.062 |
| *Elsholtzia ciliata* | 0.928 ± 0.032 | 0.770 ± 0.064 | 0.910 ± 0.049 |
| *Galinsoga parviflora* | 0.971 ± 0.006 | 0.830 ± 0.025 | 0.906 ± 0.024 |
| *Gleditsia triacanthos* | 0.929 ± 0.014 | 0.730 ± 0.033 | 0.886 ± 0.047 |
| *Hydrocotyle vulgaris* | 0.970 ± 0.005 | 0.892 ± 0.014 | 0.977 ± 0.007 |
| *Ixeridium dentatum* | 0.971 ± 0.025 | 0.902 ± 0.045 | 0.960 ± 0.022 |
| *Miscanthus sinensis* | 0.943 ± 0.017 | 0.773 ± 0.041 | 0.892 ± 0.039 |
| *Paspalum dilatatum* | 0.934 ± 0.019 | 0.753 ± 0.038 | 0.911 ± 0.024 |
| *Paulownia tomentosa* | 0.899 ± 0.021 | 0.693 ± 0.050 | 0.879 ± 0.060 |
| *Perilla nankinensis* | 0.939 ± 0.020 | 0.789 ± 0.041 | 0.917 ± 0.037 |
| *Phytolacca americana* | 0.934 ± 0.017 | 0.768 ± 0.039 | 0.927 ± 0.026 |
| *Polygonum thunbergii* | 0.953 ± 0.039 | 0.835 ± 0.089 | 0.919 ± 0.057 |
| *Pueraria lobata* | 0.928 ± 0.016 | 0.770 ± 0.035 | 0.908 ± 0.039 |
| *Robinia pseudoacacia* | 0.968 ± 0.008 | 0.842 ± 0.023 | 0.937 ± 0.016 |
| *Solidago canadensis* | 0.962 ± 0.009 | 0.813 ± 0.033 | 0.907 ± 0.021 |
| *Spiraea japonica* | 0.924 ± 0.018 | 0.770 ± 0.032 | 0.912 ± 0.032 |
| *Ulex europaeus* | 0.979 ± 0.010 | 0.890 ± 0.031 | 0.965 ± 0.010 |
| *Vitex rotundifolia* | 0.904 ± 0.067 | 0.736 ± 0.149 | 0.880 ± 0.101 |

**Table S5** Evaluation of the species distribution models evaluated using Georgian occurrences only. The table shows the mean and standard deviation of AUC, TSS and Sensitivity evaluators (see methods for more details). Only species presenting more than 12 occurrences in Georgia were evaluated. Species with good evaluations are shown in bold.

|  | **AUC** | **TSS** | **SENSITIVITY** |
| --- | --- | --- | --- |
| ***Ailanthus altissima*** | **0.741 ± 0.083** | **0.571 ± 0.118** | **0.964 ± 0.032** |
| *Ambrosia artemisiifolia* | 0.552 ± 0.038 | 0.222 ± 0.071 | 0.953 ± 0.093 |
| *Amorpha fruticosa* | NA | NA | NA |
| *Buddleja davidii* | NA | NA | NA |
| *Chenopodium album* | 0.540 ± 0.030 | 0.132 ± 0.071 | 0.775 ± 0.144 |
| *Clerodendrum bungei* | NA | NA | NA |
| ***Commelina communis*** | **0.856 ± 0.066** | **0.643 ± 0.089** | **0.896 ± 0.054** |
| *Conyza canadensis* | 0.623 ± 0.087 | 0.370 ± 0.115 | 0.959 ± 0.103 |
| ***Conyza graminifolia*** | **0.941 ± 0.033** | **0.815 ± 0.057** | **0.940 ± 0.036** |
| ***Crassocephalum crepidioides*** | **0.933 ± 0.068** | **0.849 ± 0.103** | **0.982 ± 0.045** |
| *Elsholtzia ciliata* | 0.616 ± 0.066 | 0.360 ± 0.096 | 0.597 ± 0.212 |
| *Galinsoga parviflora* | 0.650 ± 0.078 | 0.337 ± 0.111 | 0.636 ± 0.170 |
| ***Gleditsia triacanthos*** | **0.704 ± 0.116** | **0.497 ± 0.194** | **0.932 ± 0.147** |
| ***Hydrocotyle vulgaris*** | **0.891 ± 0.110** | **0.736 ± 0.240** | **0.850 ± 0.232** |
| ***Ixeridium dentatum*** | **0.931 ± 0.039** | **0.803 ± 0.073** | **0.897 ± 0.079** |
| *Miscanthus sinensis* | NA | NA | NA |
| ***Paspalum dilatatum*** | **0.881 ± 0.062** | **0.750 ± 0.069** | **0.929 ± 0.046** |
| *Paulownia tomentosa* | NA | NA | NA |
| ***Perilla nankinensis*** | **0.929 ± 0.037** | **0.793 ± 0.071** | **0.943 ± 0.040** |
| ***Phytolacca americana*** | **0.859 ± 0.054** | **0.629 ± 0.079** | **0.875 ± 0.054** |
| ***Polygonum thunbergii*** | **0.863 ± 0.037** | **0.652 ± 0.061** | **0.815 ± 0.074** |
| *Pueraria lobata* | NA | NA | NA |
| *Robinia pseudoacacia* | 0.633 ± 0.059 | 0.347 ± 0.083 | 0.957 ± 0.041 |
| *Solidago canadensis* | 0.652 ± 0.086 | 0.149 ± 0.167 | 0.803 ± 0.364 |
| *Spiraea japonica* | NA | NA | NA |
| ***Ulex europaeus*** | **0.900 ± 0.132** | **0.764 ± 0.295** | **0.941 ± 0.192** |
| *Vitex rotundifolia* | NA | NA | NA |

**Table S6** Threat potential of the 27 invasive alien plants (IAPs) for the future in Georgia, area of high plant endemism (AHPE), protected areas (PAs) and area of high conservation values (i.e. AHPE and protected areas; AHCV). Values correspond to the percent of predicted surface occupied by the different ranges of invasive species richness (0, 1-9, 10-18 and 19-26 species). Future predictions are presented for three different climate change scenarios for the year 2050: RCP 4.5 HadGEM2-AO, RCP 4.5 IPSL-CM5A-LR and RCP 8.5 HadGEM2-AO.

**RCP 4.5 HadGEM2-AO**

| IAPs richness | Georgia (%) | AHPE (%) | PAs (%) | AHCV (%) |
| --- | --- | --- | --- | --- |
| 0 | 7.61 | 0.31 | 17.57 | 5.67 |
| 1-9 | 36.31 | 17.40 | 33.07 | 22.37 |
| 10-18 | 45.45 | 66.00 | 36.31 | 57.31 |
| 19-26 | 10.62 | 16.29 | 13.04 | 14.64 |

**RCP 4.5 IPSL-CM5A-LR**

| IAPs richness | Georgia (%) | AHPE (%) | PAs (%) | AHCV (%) |
| --- | --- | --- | --- | --- |
| 0 | 3.20 | 0.32 | 9.29 | 3.12 |
| 1-9 | 34.60 | 14.92 | 36.75 | 21.69 |
| 10-18 | 47.94 | 58.65 | 36.37 | 51.85 |
| 19-26 | 14.24 | 26.12 | 17.60 | 23.34 |

**RCP 8.5 HadGEM2-AO**

| IAPs richness | Georgia (%) | AHPE (%) | PAs (%) | AHCV (%) |
| --- | --- | --- | --- | --- |
| 0 | 4.43 | 0.40 | 4.41 | 1.66 |
| 1-9 | 37.36 | 16.71 | 43.40 | 25.16 |
| 10-18 | 50.75 | 66.96 | 41.30 | 59.34 |
| 19-26 | 7.44 | 15.93 | 10.90 | 13.84 |

**Figure S1** Occurrences (black points) of the endemic plant species in Georgia, and location of the areas of high plant endemism (black-rimmed frames) and PAs (grey-rimmed frames).
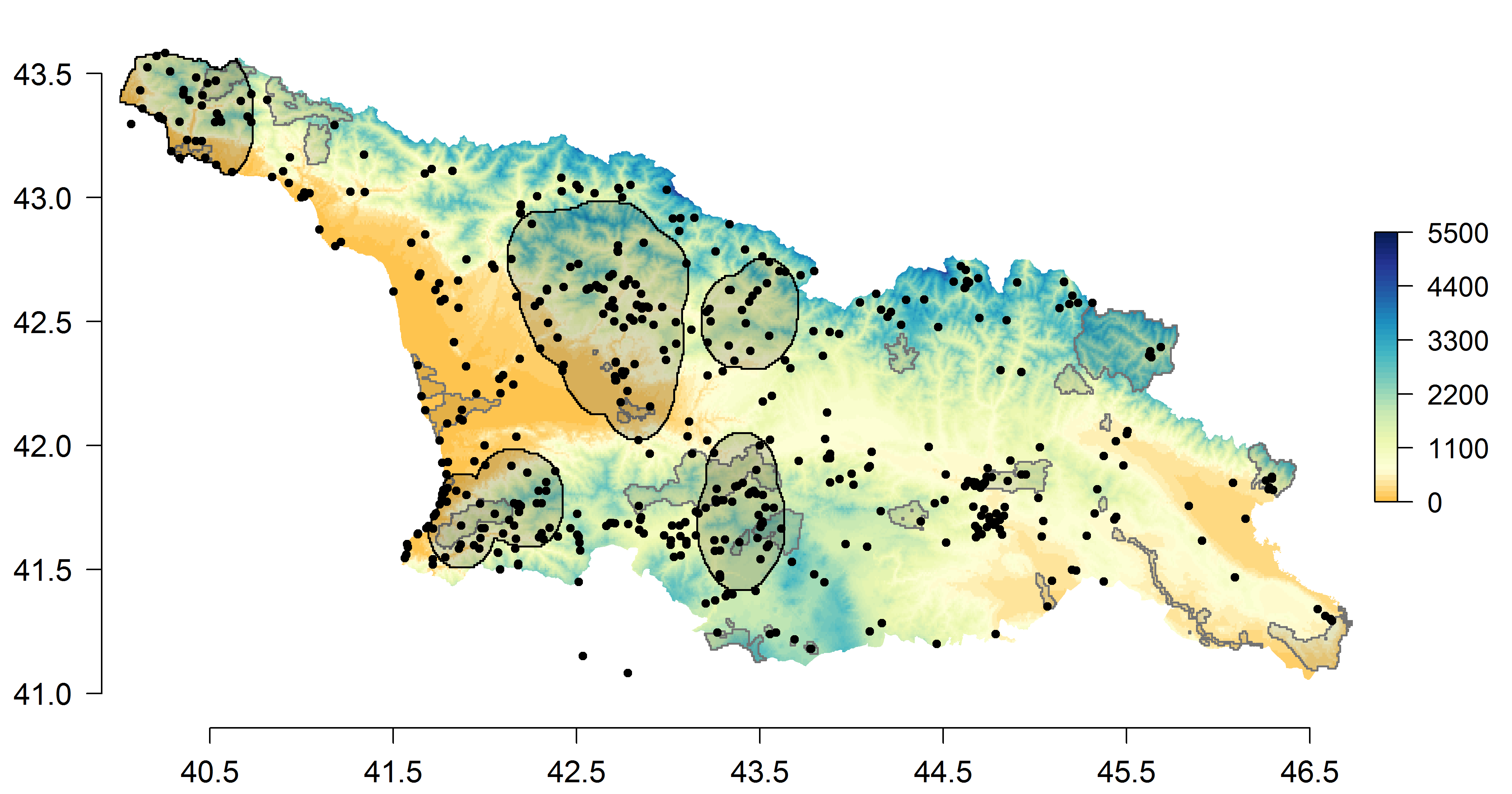


**Figure S2** Occurrences (black points) of the endemic plant species in Georgia, and location of the areas of high plant endemism representing 20% of the total cover of Georgia (grey area; > 15 endemic species), 10% (yellow area; >= 18 endemic species), 5% (orange area; > 22 endemic species) and 1% (red area; > 26 endemic species).
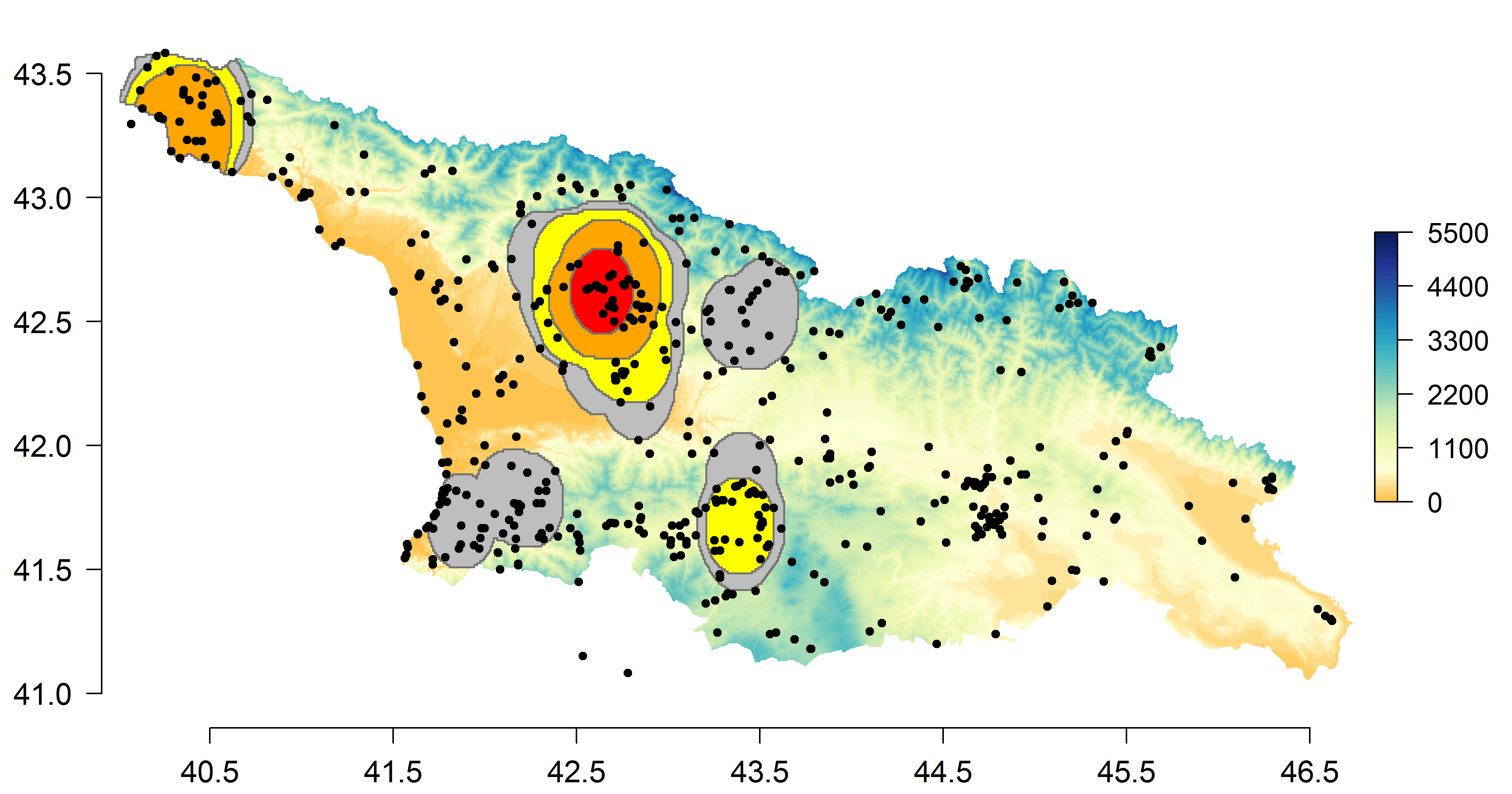


**Figure S3** Distribution of the occurrences of the 27 invasive alien plant species (red points) in the world.


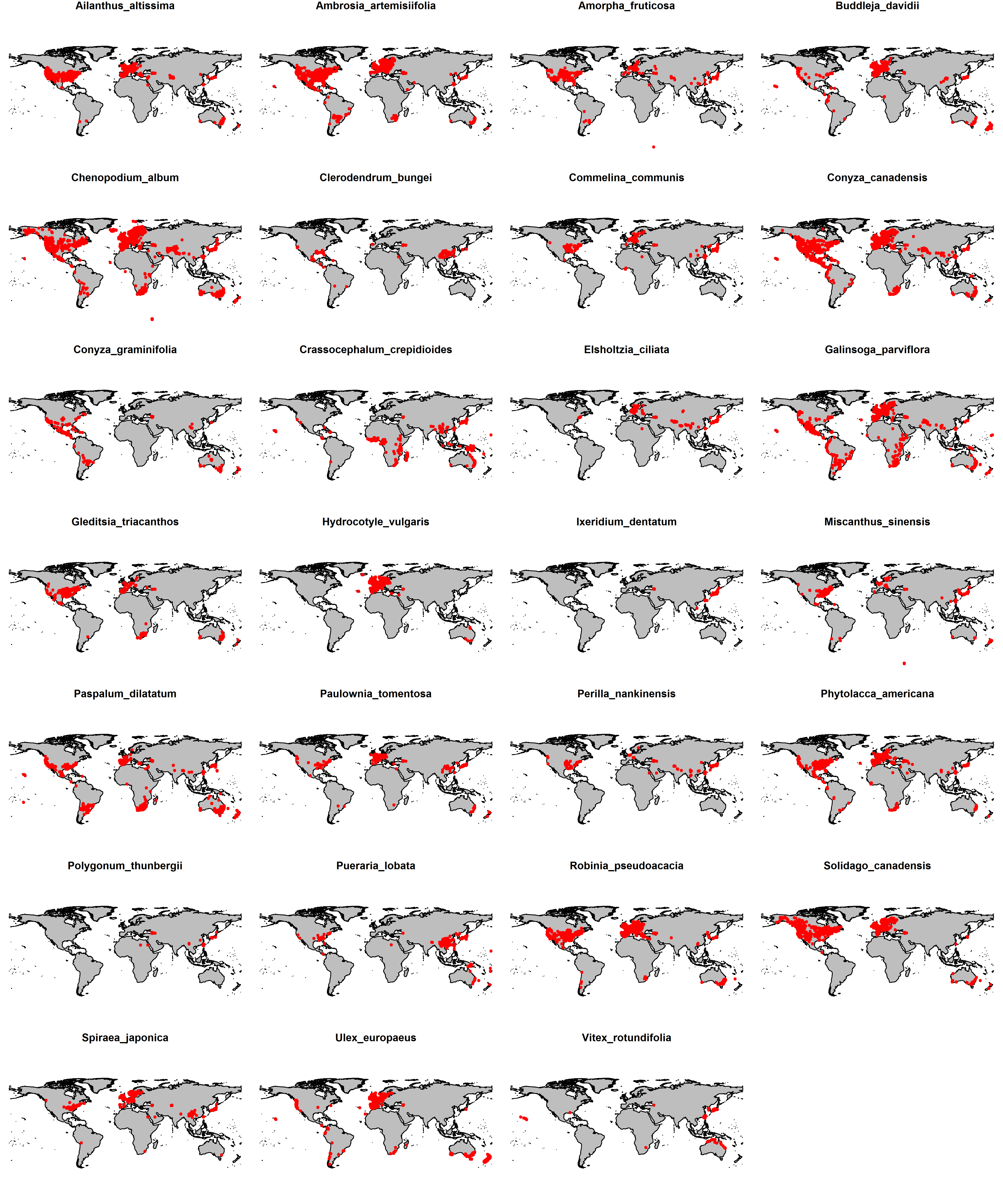


**Figure S4** Occurrences (black points) of the 27 selected invasive alien plant species in Georgia, and location of the areas of high plant endemism (black-rimmed frames) and PAs (grey-rimmed frames).

**
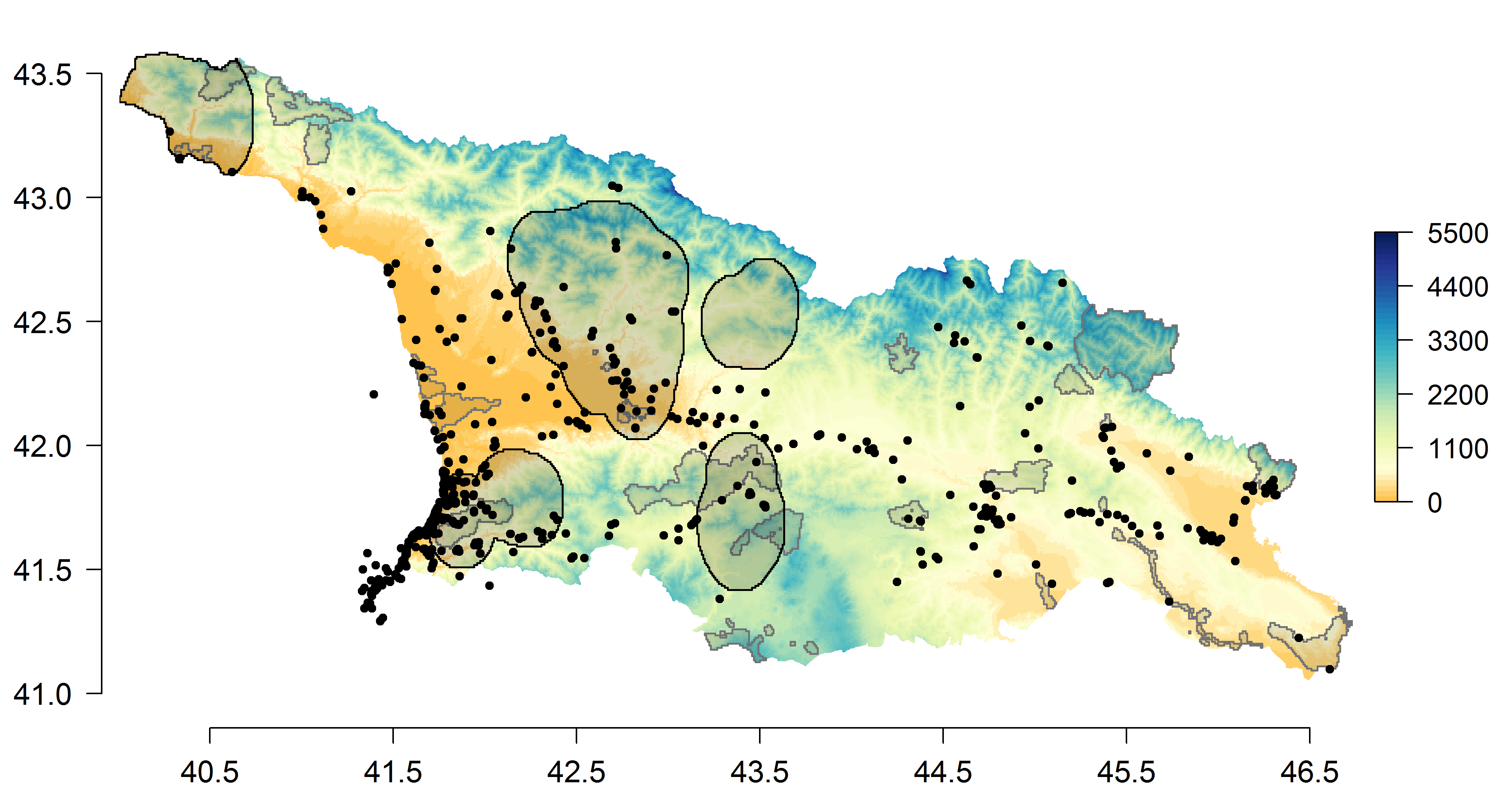
**

**Figure S5** Summed species mean predictions of habitat suitability (mean of the 5 algorithms) for invasive alien plant species in Georgia for the (a) present climate and (b) future climate for the year 2050 (RCP 8.5 IPSL-CM5A-LR climate change scenario). The protected areas are shown as grey-shaded frames and areas of high plant endemism as black-rimmed frames. The colour scale represents the summed mean predictions of habitat suitability for invasive alien plant species in Georgia.

**
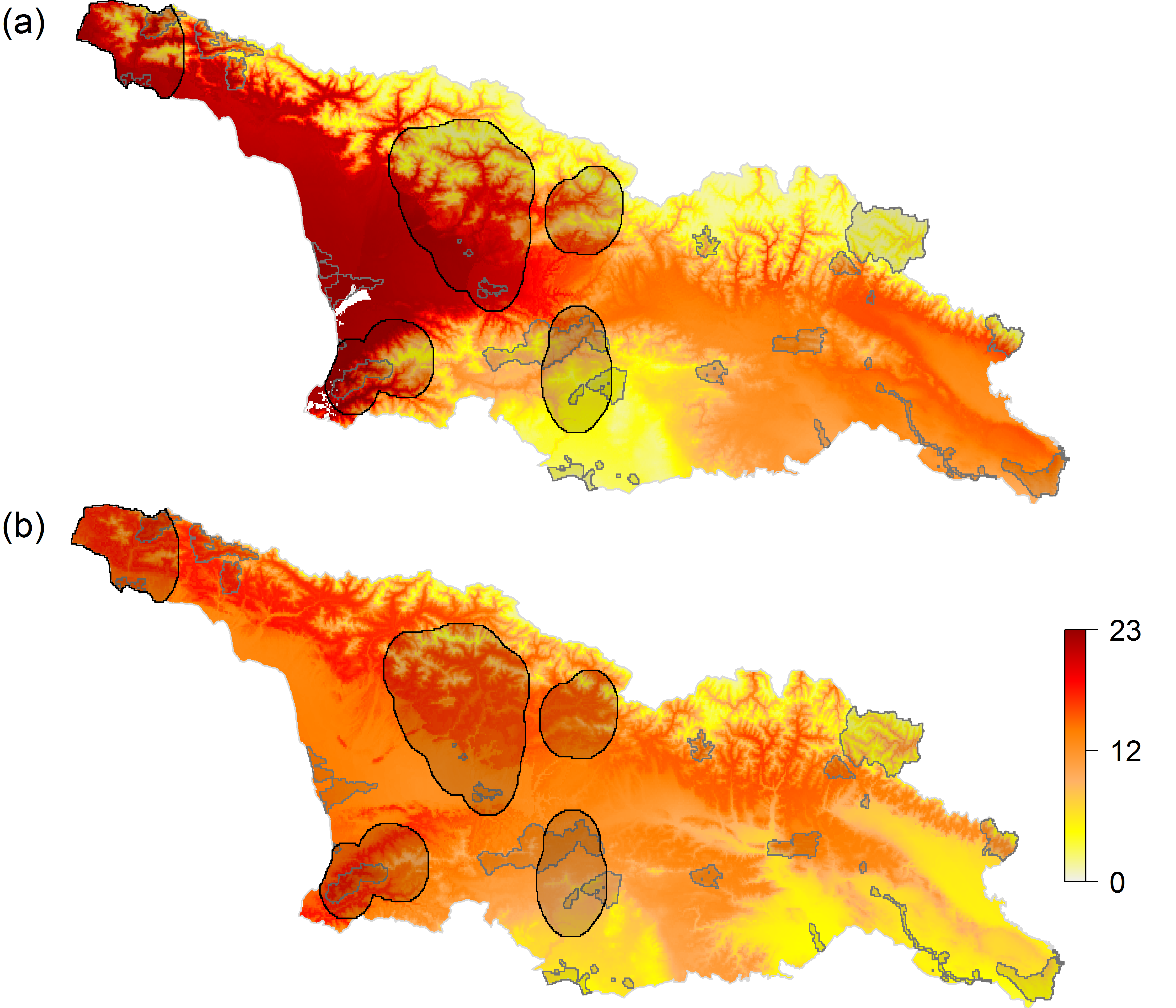
**

**Figure S6** Species mean predictions of habitat suitability (mean of the 5 algorithms; green = high suitability; white=low suitability) for invasive alien plant species in Georgia with their occurrences in the country (black points).


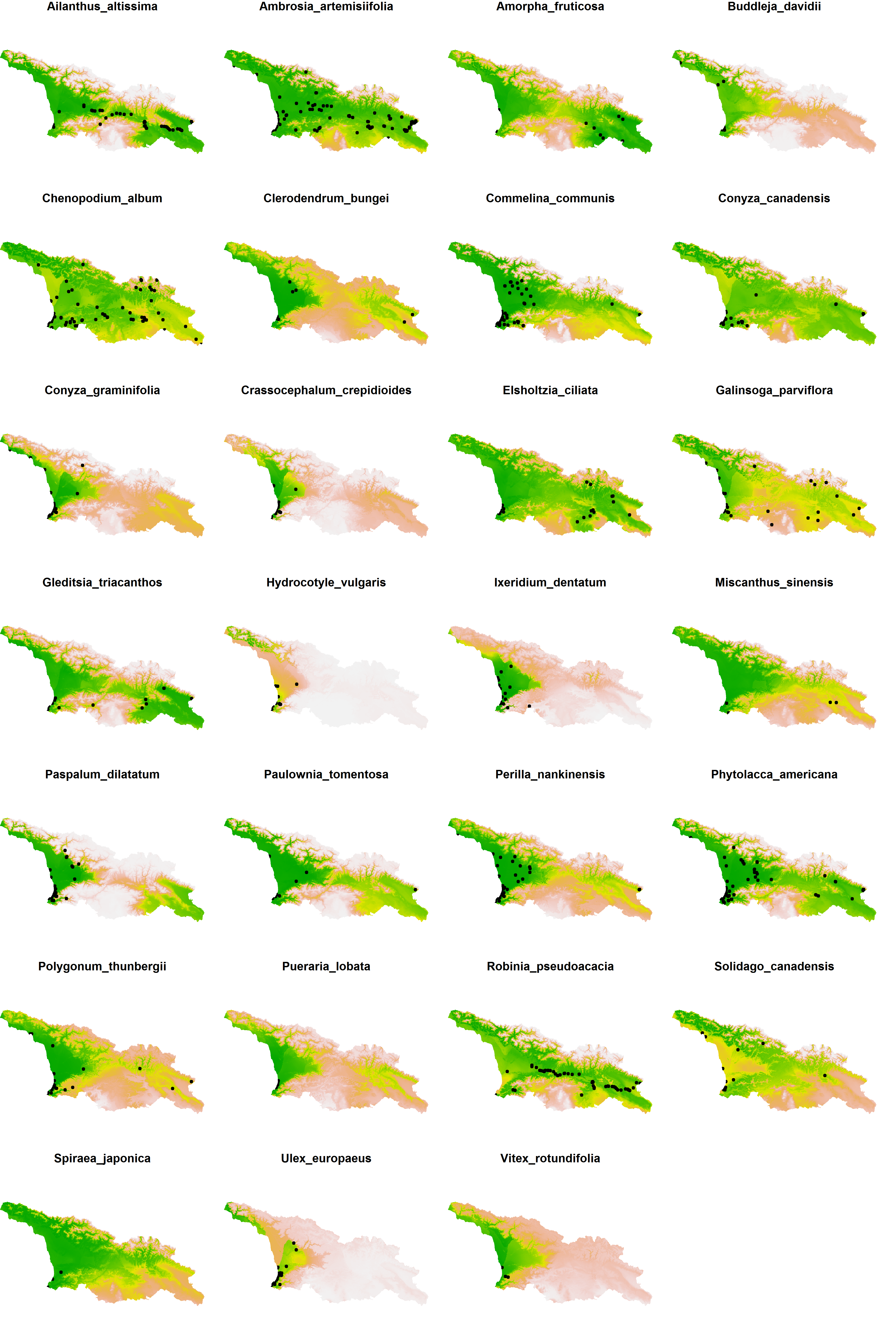


**Figure S7** Predicted current distribution (green area) of invasive alien plant species in Georgia with their occurrences in the country (black points).

**
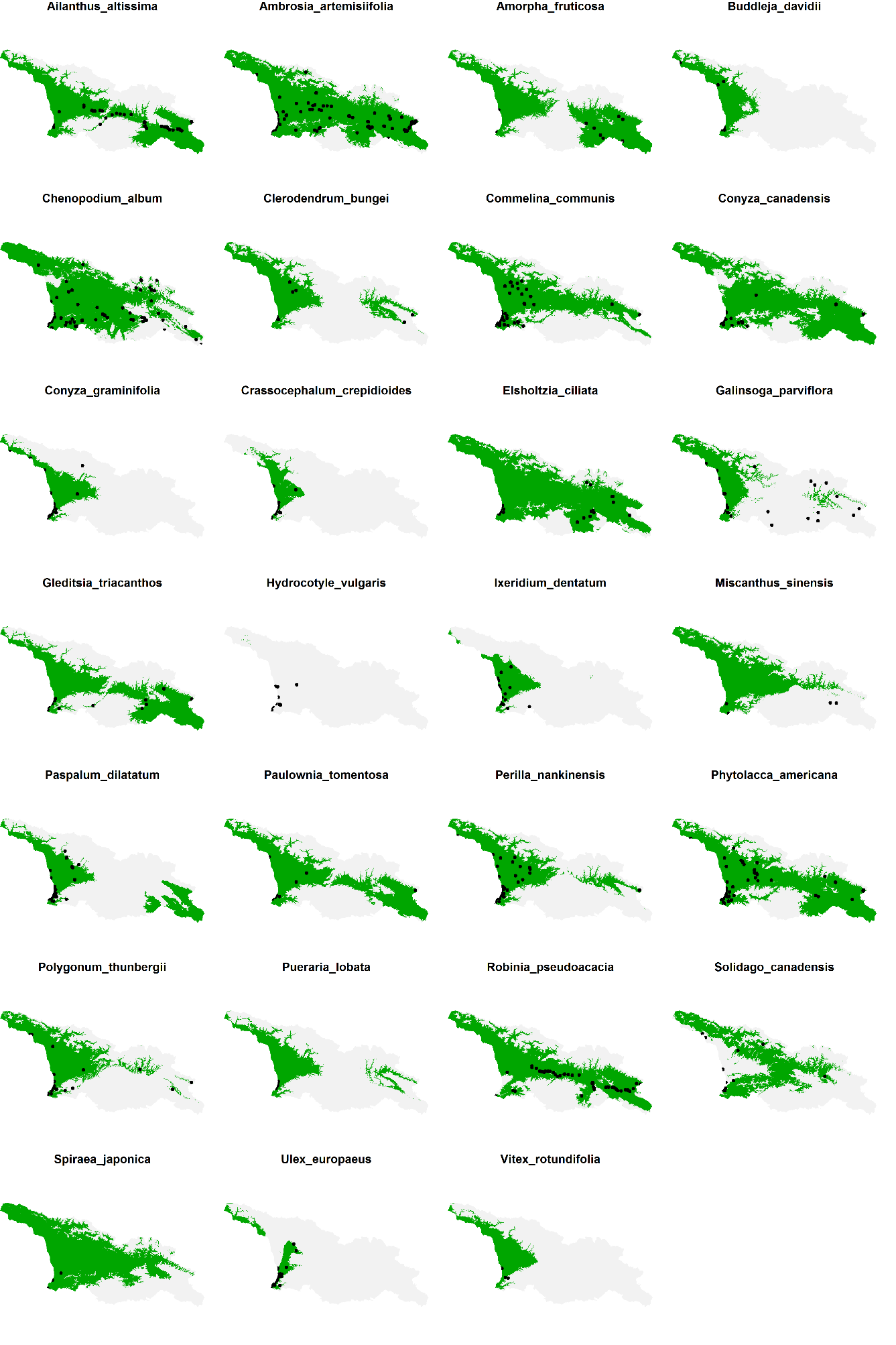
**

**Figure S8** Invasive alien plant species richness in Georgia for future climate under three different climate change models for the year 2050: (a) RCP 4.5 HadGEM2-AO, (b) RCP 4.5 IPSL-CM5A-LR and (c) RCP 8.5 HadGEM2-AO.


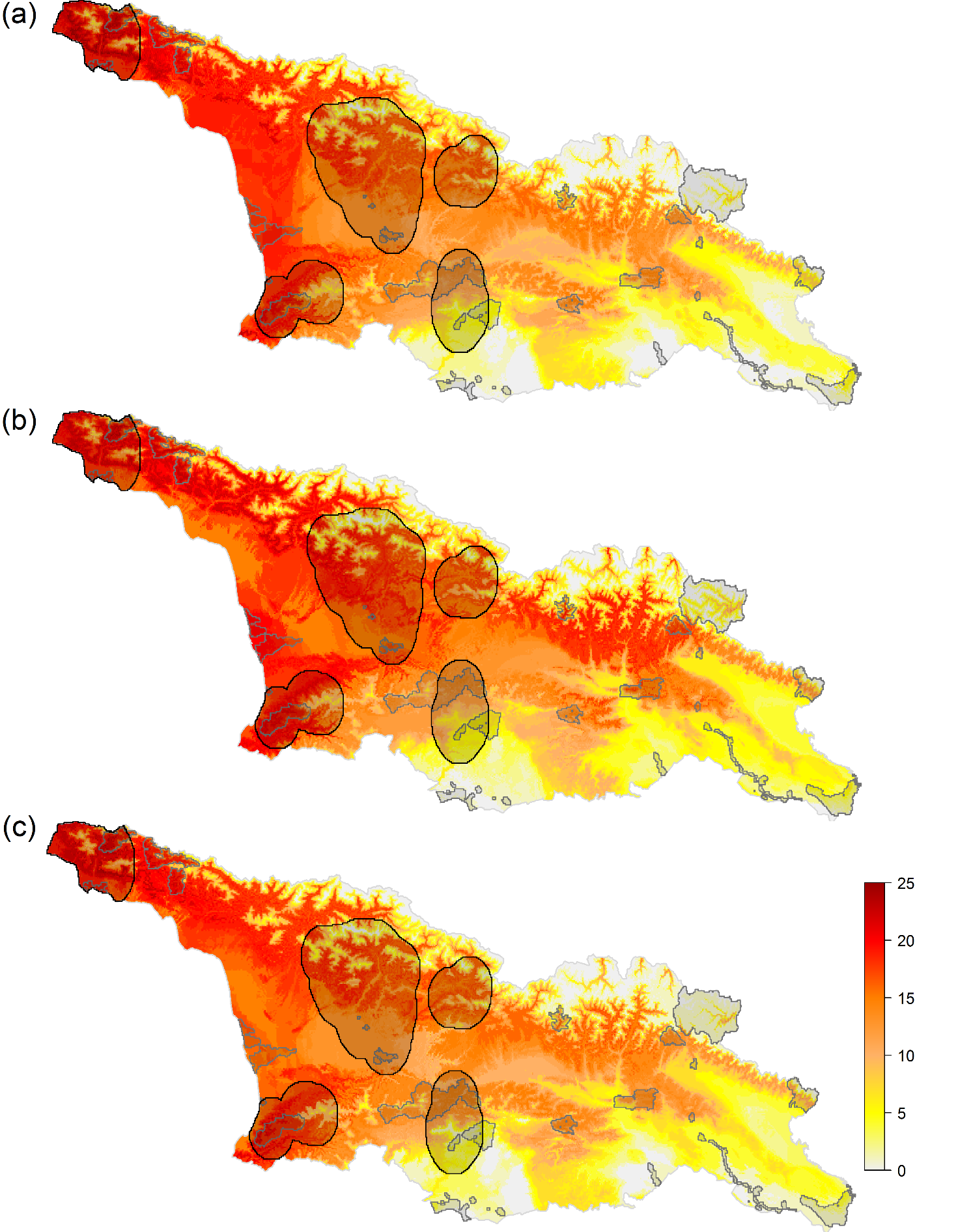

Supplement: Supplementary file 1 [file ECE3-8-4431-s001.docx]
